# Succession of microbial community composition and secondary metabolism during marine biofilm development

**DOI:** 10.1093/ismeco/ycae006

**Published:** 2024-01-20

**Authors:** Pernille Kjersgaard Bech, Scott A Jarmusch, Jacob Agerbo Rasmussen, Morten Tønsberg Limborg, Lone Gram, Nathalie N Suhr Eiris Henriksen

**Affiliations:** Department of Biotechnology and Biomedicine, Technical University of Denmark, Kgs. Lyngby, DK-2800, Denmark; Department of Biotechnology and Biomedicine, Technical University of Denmark, Kgs. Lyngby, DK-2800, Denmark; Center for Evolutionary Hologenomics, GLOBE Institute, University of Copenhagen, Copenhagen K, DK-1014, Denmark; Center for Evolutionary Hologenomics, GLOBE Institute, University of Copenhagen, Copenhagen K, DK-1014, Denmark; Department of Biotechnology and Biomedicine, Technical University of Denmark, Kgs. Lyngby, DK-2800, Denmark; Department of Biotechnology and Biomedicine, Technical University of Denmark, Kgs. Lyngby, DK-2800, Denmark

**Keywords:** community succession, biosynthetic gene clusters, secondary metabolites, marine microbial communities, biofilm, metagenomics, untargeted metabolomics

## Abstract

In nature, secondary metabolites mediate interactions between microorganisms residing in complex microbial communities. However, the degree to which community dynamics can be linked to secondary metabolite potential remains largely unknown. In this study, we address the relationship between community succession and secondary metabolism variation. We used 16S and 18S rRNA gene and adenylation domain amplicon sequencing, genome-resolved metagenomics, and untargeted metabolomics to track the taxons, biosynthetic gene clusters, and metabolome dynamics *in situ* of microorganisms during marine biofilm succession over 113 days. Two phases were identified during the community succession, with a clear shift around Day 29, where the alkaloid secondary metabolites, pseudanes, were also detected. The microbial secondary metabolite potential changed between the phases, and only a few community members, including *Myxococotta* spp., were responsible for the majority of the biosynthetic gene cluster potential in the early succession phase. In the late phase, bryozoans and benthic copepods were detected, and the microbial nonribosomal peptide potential drastically decreased in association with a reduction in the relative abundance of the prolific secondary metabolite producers. Conclusively, this study provides evidence that the early succession of the marine biofilm community favors prokaryotes with high nonribosomal peptide synthetase potential. In contrast, the late succession is dominated by multicellular eukaryotes and a reduction in bacterial nonribosomal peptide synthetase potential.

## Introduction

A central focus of microbial ecology is to determine the forces that govern structural and functional changes of microbial communities, including assembly and succession dynamics found in microbial biofilms [1-5]. However, it is only recently that microbial chemical ecologists have begun to acknowledge the many roles microbial secondary metabolites may play in microbial communities [6]. In fact, the term “keystone metabolites” was recently used to describe the potential power of these complex molecules in not only microbial communities but also whole ecosystems [7]. Thus, to determine the full functionalities of microbial communities and their many different roles, it is essential to understand if and how microbial secondary metabolites shape their development. This implies that a temporal scale must be included in such studies.

Microbial secondary metabolites play a role in several microbial interactions and are important modulators of microbial behavior [8]. They can mediate interference competition, virulence, and nutrient scavenging and are produced by a multitude of microorganisms [9-11]. Much of our understanding of secondary metabolites and microbial community processes has been gained from pure culture or *in vitro* model systems. While providing essential knowledge about single-handed mechanisms that govern communities (i.e. colonization patterns, interspecies interactions, and phenotypic traits), such studies are constrained to narrow phylogenetic groups and/or performed under conditions far away from the ecological context [12]. In contrast, large-scale field studies are still limited, probably not only due to the lack of experimental control in complex natural ecosystems but also due to technology still not allowing the dissection of molecular mechanisms directly in nature. However, the understanding obtained from model organisms on molecular mechanisms of secondary metabolites and the increasing quality of computational tools has allowed us to use genomic traits to predict the biosynthetic gene cluster (BGC) potential, allowing predictions on how microorganisms cope with environmental conditions [13]. Most *in situ* studies on secondary metabolites have focused on profiling the BGC potential of different environments for bioprospecting purposes [14-17]. Efforts within this research area recently revealed that secondary metabolites co-occur in a habitat-specific manner, thus facilitating the distinction of environments on Earth [18]. However, these large-scale omics studies only provided “snapshots” of the microbial communities and secondary metabolite potential, hence neglecting the importance of microbial community intrinsic processes and successional changes. One may assume that fluctuations in environmental conditions and community composition will lead to changes in the presence of secondary metabolites. To the best of our knowledge, only one study has examined the changes in the chemical composition of a microbial community through ecological succession [19]. Therefore, studies examining the genetic and metabolic response of the microbial communities to different successional phases are scarce.

Here, we analyzed the genetic potential for microbial secondary metabolite production during microbial community assembly *in situ* and succession in a marine biofilm community. We used a combination of targeted amplicon and genome-resolved metagenomics. We further combined the metagenomic results with metabolomic analyses to track functional dynamics during the succession of a marine community. Besides the fundamental biological understanding, such studies of temporal dynamics can guide more applied bioprospecting, indicating not only the sites but also the times at which the discovery of novel compounds is most likely at a given site.

## Results

**Figure 1:**
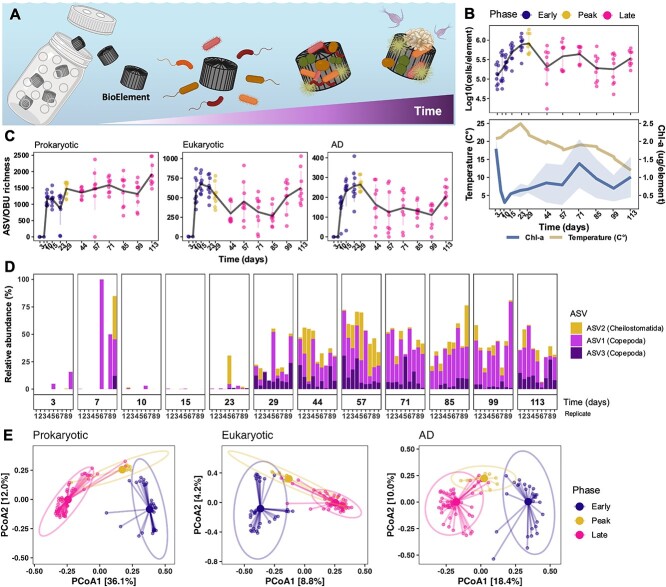
Microbial community dynamics during marine biofilm succession; (A) experimental setup; nine plastic cages containing plastic elements (BioElements) were submerged in coastal seawater and sampled over four months; at each sampling time point, one plastic element was collected, one from each of the nine plastic cages, to represent the microbial biofilm community at the given time point; illustration is created with Biorender.com; (B) the upper plot shows the absolute abundance of microbial cells (cell size <100 μm) in the collected microbial biofilms; mean and SD are shown as a solid line and error bars, respectively; lower plot shows the surrounding seawater temperature and the mean chlorophyll-*a* concentration over time; ribbons represent SD; *N* = 9. (C) 16S, 18S, and AD observed richness over time in the biofilm communities; mean and SDs are shown; each point corresponds to one sample and is colored by the respective phase defined in (B); *N* = 2–9; (D) relative abundance of top three eukaryotic ASVs per time point in the eukaryotic communities; *N* = 2–9; (E) community compositions visualized as Bray–Curtis distances in PCoA ordinations; samples are connected to the centroid of the corresponding phase; from left: 16S (*n* = early: 27, peak: 9, late: 52), 18S (*n* = 31, 9, 53), and ADs (*n* = 34, 9, 55); ellipses represent 95% confidence intervals.

We developed an *in situ* system to study microbial community development in a marine biofilm on solid surfaces (BioElements) in a coastal seawater environment. These BioElements were enclosed in nine separated plastic cages; thus, BioElements were taken from each of the nine plastic cages at each time point (Fig. 1A and Supplementary Fig. S1A). We chose to investigate marine microbial biofilms because the emergent properties of microbial biofilms, such as structural rigidity, facilitate microbial interactions and, therefore, have the potential to facilitate microbial secondary metabolite production [20, 21]. This *in situ* system allowed us to link the bacterial secondary metabolism dynamics and the community composition over spatially distinct but temporally synchronized BioElements over four months from June to October. At discrete time intervals, we collected BioElements from each cage (one for chlorophyll-*a*, one for flow cytometry, one for metabolomics, and one for DNA) to assess the temporal dynamics of the biofilm community composition, diversity, BGC profiles, and metabolome.

### The temporal abundance of the biofilm microbial community

We quantified the number of microbial cells (cell size <100 μm) detached from the BioElements using flow cytometry (Fig. 1B) to characterize the total microbial abundance and succession dynamics in the microbial communities. Across nine BioElement replicates, the dynamics roughly followed a logistic growth model where the abundance was positively correlated with temperature (spearman rho = 0.37, *P*-value <.001). In particular, at day 3, the microbial community cell abundance was estimated to mean ± standard deviation (SD) log 5.1 ± 0.3 cells/BioElement, whereafter it increased rapidly until day 29, where it peaked with log 5.9 ± 0.2 cells/BioElement. Subsequently, the microbial cell abundance significantly decreased (Dunnett’s test; *P*-values <.01) and remained below the cell abundance observed from the earlier time points. Based on the temporal patterns in cell abundances, we identified three discrete phases of community succession. In the first phase (Early; day = 3–23), the microbial community steadily increased in absolute abundance. In the second phase (Peak; day = 29), the microbial community “peaked” in cell abundance. The third phase (Late; day = 44–113) was characterized by a decline in microbial cell abundance from day 29 to 44, followed by variable, but relatively low abundances that reached a minimum at day 99 at log 5.25 ± 0.4 cells/BioElement.

We measured chlorophyll-*a* concentrations as a biomass measure over time to account for photosynthetic organisms on the BioElements (Fig. 1B). The highest chlorophyll-*a* concentration at 1.8 ± 03 μg/BioElement was measured 3 days after immersion. It was followed by a steep decrease until day 10, suggesting the rapid loss of primary colonizing photosynthetic microorganisms. Over the next two months (days 10–71), the mean chlorophyll-*a* concentration increased, resulting in a significant difference between day 10 and day 71 (Wilcoxon test: *P*-value <.01), thus indicating the increase of photosynthetic biomass over time. However, the SD increased over time, indicating increased variation among replicates (Fig. 1B).

### Taxonomic composition patterns during community succession

To assess the prokaryotic and eukaryotic community compositions and diversities over time, we used amplicon sequencing of the 16S and 18S rRNA genes to generate amplicon sequencing variants (ASVs). From the immersion of the BioElements, the prokaryotic ASV richness increased and reached a plateau at day 29 with 1477 ± 215 ASVs (Fig. 1C). The richness remained within this level in the remaining time with the highest richness reached at day 113 with 1928 ± 367 ASVs (Fig. 1C). In contrast, the eukaryotic richness decreased significantly from Days 23 to 44 (Tukey’s test: *P*-value <.01, Fig. 1C). During this decrease, colonization of the bryozoan *Conopeum seurati* (order: *Cheilostomatida*; visual identification based on its morphological features, Supplementary Fig. S1B and C) was observed by the naked eye (Supplementary Fig. S1B). The bryozoan colonization was also reflected in the eukaryotic ASV composition, where a few ASVs, namely, ASV1/ASV3 (class: *Copepoda*, order: *Harpacticoid*) and ASV2 (Phylum: *Bryozoa*, order: *Cheilostomatida*), increased rapidly in relative abundance after day 29 (Fig. 1D). This trend could also be observed by decreased Shannon diversity after day 29, due to a skewed evenness (Supplementary Fig. S2A). This decline in the eukaryotic diversity and the arrival of the bryozoans along with the copepods indicate a successional shift between Days 29 and 44. This successional shift in the eukaryotic fraction co-occurred with the decrease in microbial cell abundance (<100 μm). This co-occurrence could suggest a shift from “bottom–up” to “top–down” control as previously described [3].

A total of 14.3% of the prokaryotic and 11.3% of the eukaryotic compositional variation were described by the three previously defined community phases (Permutational multivariate analysis of variance [PERMANOVA], *P*-values <.001), and this was confirmed by the clustering in the principal coordinate analysis (PCoA) (Fig. 1E). Within the early and peak phase for the prokaryotic and eukaryotic communities, the communities clustered clearly according to discrete time points, in contrast to the late phase, where the communities converged (Supplementary Fig. S3). Additionally, the eukaryotic community composition variances within the peak and late phase were significantly larger compared to the early phase (beta-dispersion; *P*-value <.001; Tukey’s *t*-test; *P*-values <.0001), while the dispersion within the two phases and the peak for the prokaryotic community compositions was similar (beta-dispersion; *P*-value = .474). This could suggest that the eukaryotic communities were more susceptible to stochasticity (e.g. temporal and/or environmental changes). Thus, the communities became more distinct from each other over time compared to the prokaryotic communities. Stochastic fluctuations throughout time and genetic variation (drift and diversification) are inherent properties of time-dependent community studies that commonly lead to divergent or convergent communities [22-24].

Since the biofilm communities originated from spatially distinct replicates (maximum ~3 m apart, Supplementary Fig. S1A), spatial location (replicate) could be responsible for the variation observed in community compositions. However, replicates only explained a minor part of the eukaryotic community composition (R2 = 0.01443, *P*-value = .031) and none of the prokaryotic community compositions (*P*-value = .091). On the other hand, a significant association was found between the prokaryotic and the eukaryotic composition supported by the Procrustes analysis (M2 = 0.303, *P*-value = .001).

### The early succession phase of the biofilm community possesses a higher nonribosomal peptide potential

To assess temporal changes in the biosynthetic potential of the microbial biofilm communities over time, we used an amplicon-based approach targeting the adenylation domains (ADs) in the nonribosomal peptide synthetase (NRPS) genes followed by clustering into 99% operational biosynthetic units (OBUs) [14, 25].

Overall, the biosynthetic potential was higher in the biofilm communities compared to the surrounding seawater both during the early and peak phase (Linear Mixed Model [LMM] and estimated marginal means [EMMs] = 110, *P*-values <.001, Supplementary Fig. S2B). In the late phase, the biosynthetic potential of the biofilm communities had a higher variability (Tukey multiple comparisons of means; *P*-values <.001) than in the peak and early phase. This reflected the bacterial compositional variance across time supported by Procrustes analysis (M2 = 0.359, *P*-value = .001) and the clustering of the AD OBU composition in the PCoA ordination (Fig. 1E). Interestingly, the AD OBU richness pattern in the developing biofilm did not follow that of the prokaryotic richness (Fig. 1C). This was further supported by the fact that the AD OBU richness was not correlated with the prokaryotic richness (Spearman correlation, rho: 0.02, *P*-value = .83, Supplementary Fig. S2C).

We hypothesized that the NRPS BGC potential would positively correlate with prokaryotic richness. However, since we did not observe this correlation (Supplementary Fig. S2C), we speculated that the increasing AD OBU richness within the early to peak phase could be explained by a few prokaryotic taxa having a high NRPS potential, and that these were reduced or eliminated from the community during the late phase, hence explaining the significant drop in the AD OBU richness between days 29 and 44–99 in the late phase (Dunnett’s test; *P*-values <.03). Indeed, several ASVs belonging to the *Proteobacteria*, *Bdellovibrionota*, *Planctomycetota*, and *Verrucomicrobiota* disappeared between peak and late phase (days 44–57), where especially ASVs belonging to the phylum *Myxococcota*, *Actinobacteriota*, *Spirochaetota*, and *Fusobacteriota* were a pronounced part of the peak phase (Supplementary Fig. S4). Several members within these phyla, especially *Actinobacteria* and *Myxobacteria*, have a large biosynthetic potential [26, 27]. However, the direct linkage of AD OBUs to prokaryotic ASVs is fundamentally not feasible.

### Few taxa were responsible for the majority of the nonribosomal peptide synthetase potential

To determine if particular taxa in the biofilm community could be linked to the NRPS biosynthetic potential, shotgun metagenomes were generated to link functionality to taxonomy. Shotgun-sequenced metagenomes were generated using coassembly from eight samples originating from eight different days (days 10, 15, 23, 29, 44, 71, 85, 99, and 113), resulting in 200 Gb of raw sequence data. The co-assembly consisting of the nine samples resulted in 444 232 contigs, with an N50 of 5295 bp and more than 2 124 856 open reading frames (ORFs). Saturation curves of ORFs found in the coassembly, particularly the samples from the early phase, were flattening (Supplementary Fig. S5A), which we suggest is related to low biodiversity in the early phase leading to higher coverage per bacterial genome rather than differences in total sequencing output. To estimate the completion of metagenome-assembled genomes (MAGs), we used the method of single-copy core genes (SCGs) following the Anvi’o pipeline (https://anvio.org/). The abundance of 71 SCGs indicated the potential to assemble 340 MAGs, which matched well with the 306 that were recovered (Supplementary Fig. S5B).

A total of 17.5% of the 2 209 unique AD OBUs were mapped to the 306 MAGs with identity >95% and query length > 200 bases, whereas 34.4% of the AD OBUs could be mapped to the full coassembly (Supplementary Fig. S5C). We considered if the unmapped AD OBUs (~65.5%) were low-abundant OBUs and therefore not represented in the MAGs due to low sequencing effort. However, the relative abundance of AD OBUs was equally distributed across both high- and low-abundant AD OBUs (Supplementary Fig. S5C), suggesting that an unknown fraction of the AD OBUs could be artificial variants generated during the inference of the OBUs or due to Polymerase chain reaction (PCR) biases. Consequently, the amplicon-based method may overestimate the true complexity of AD domains in the marine biofilm (Supplementary Fig. S5C).

**Figure 2:**
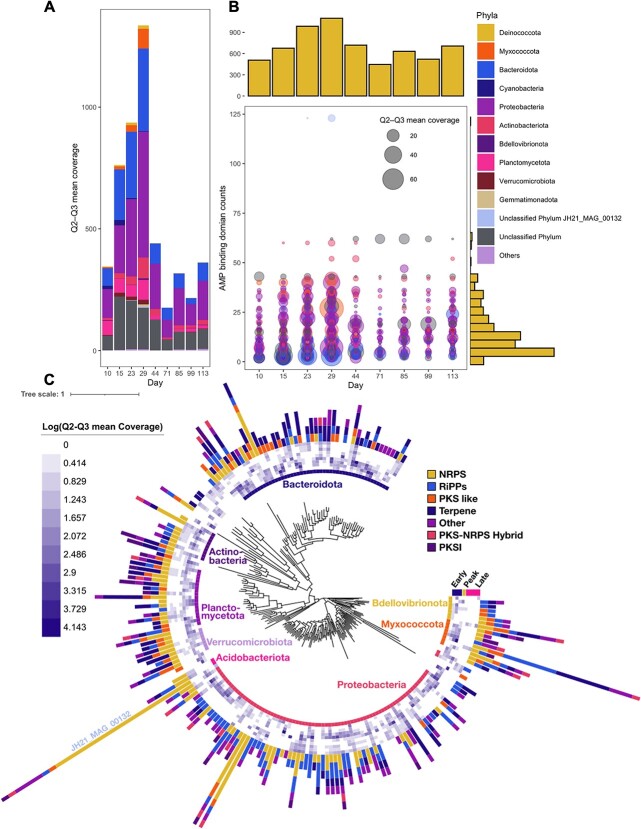
MAGs found in the marine microbial biofilm community across time; (A) mean coverage abundance of curated MAGs from the coassembly, constituting a subset of samples from 9 days (Days 10, 15, 23, 29, 44, 71, 85, 99, and 113); color indicates phylum classification; *N* = 1; (B) bubble plot with distribution of AMP-binding domains with total counts of annotated MAGs across time; each bubble represents one MAG colored by phylum annotation; size relates to mean coverage abundance at each time point; the horizontal histogram indicates the sum of AMP-binding domains per time point, and the vertical histogram indicates the distribution of AMP-binding domains per MAG; (C) phylogeny of the recovered MAGs; inner layer: phylum classification; second layer: heatmap indicating the mean coverage abundance of the MAGs across time; outer layer: antiSMASH predicted BGC counts colored by BiG-SCAPE class.

To identify MAGs with genes encoding AD domains, we targeted the AMP-binding domains homologous to AD domains [28]. In the coassembly, 5 696 ORFs were annotated as AMP-binding domains, and of these, 3655 AMP-binding domains were allocated among the curated MAGs, with the largest proportion found in the peak phase (Day 29; Fig. 2B), corresponding well with the AD amplicon–predicted richness (Fig. 2C). The majority of MAGs had between 2 and 20 AMP-binding domains, yet we found one MAG (JH21_MAG_00132) belonging to an unknown phylum, with 80% completeness, a genome size of 5.65 Mbases, and an N50 of 6217 bases, that contained a total of 123 AMP-binding domains (Fig. 2A and B). This MAG reached 5.41 × Q2–Q3 mean coverage in the peak phase, together with four other MAGs classified as *Myxococcota* (JH21_MAG_00014, JH21_MAG_00071, JH21_MAG_00065, JH21_MAG_00094), which contained more than 20 AMP-binding domains per MAG but were completely absent after the peak phase (day 29; Fig. 2A). In conclusion, this suggested that specific prolific NRPS producers were disappearing in the late phase.

### High abundance of secondary metabolite genes and stress genes characterize the early-phase biofilm

Next, we analyzed the broader BGC potential in the MAGs. The analysis confirmed that the high number of AD domains found in JH21_MAG_00132 and the myxobacteria (JH21_MAG_00014, JH21_MAG_00071, JH21_MAG_00065, JH21_MAG_00094) was associated with NRPS BGCs (Fig. 2C). Besides this, any of these MAGs also had a high genomic potential to produce RiPPs, terpenes, and PKS-like compounds that were significantly allocated to the early/peak phase of the succession in contrast to MAGs with low abundance in the late phase (Fig. 2C).

**Figure 3:**
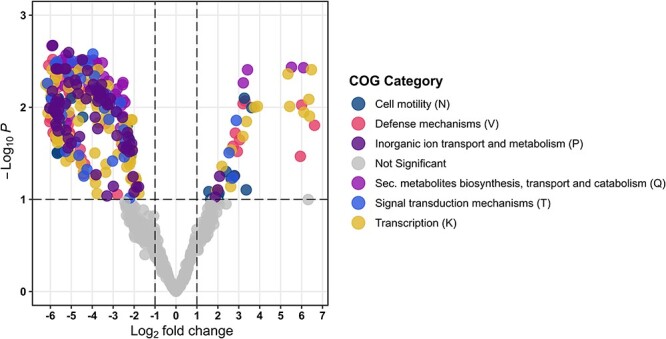
Functional analysis of stress-related genes; differential abundance of functional stress-related genes; genes with negative Log_2_ fold change were more abundant in the early-peak phase (*n* = 4), and genes with positive Log_2_ fold change were more abundant in the late phase (*n* = 5); each point represents a functional gene, colored by COG category.

Besides the BGC potential, the functional genomic potential varied between the early/peak and late phase within specific Clusters of Orthologous Groups (COG) categories (Fig. 3). Differential abundance analysis of the coassembly data revealed that genes encoding defense mechanisms, such as *emrA* and *ercB* encoding multidrug efflux pump [29, 30] and *salY* encoding ABC-type antimicrobial peptide transport system [31], were significantly more abundant in the early-peak phase (adjusted *P*-value <.05) (Fig. 3). Accordingly, genes related to secondary metabolisms were significantly enriched in the early-peak phase. Specifically, *bstA*/*dinB*, encoding *Bacillithiol/mycothiol* S-transferase [32, 33], and *hutI*, encoding a part of the imidazolonepropionase or a related amidohydrolase biosynthesis [34], were enriched in this period. Lastly, prokaryotic stress defense genes, such as genes encoding outer membrane receptor protein for iron transport (CirA) [35] and genes associated with the universal stress protein (UspA) [36], were also significantly more enriched in the early-peak phase.

### The temporal metabolic landscape and detection of secondary metabolites

**Figure 4:**
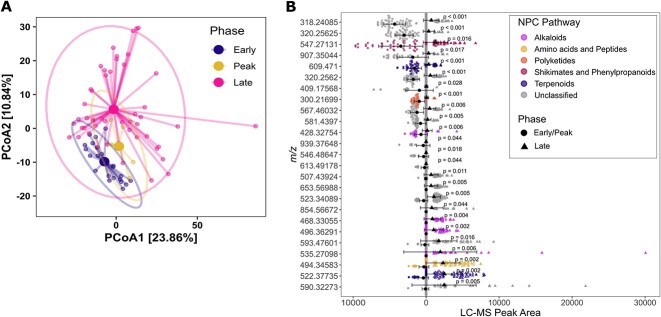
Ordination and differential analysis of the metabolomic LC-MS features; (A) metabolome composition of features >300 *m/z* visualized as Euclidean distances in PCoA ordinations; ellipses represent 95% confidence intervals; each sample is connected to the centroid of the corresponding phase (early, peak, or late); *N* = 23, 8, and 50; (B) differential analysis of metabolic features dominating the early/peak (*n* = 29) vs. late phase (*n* = 49) (Kruskal–Wallis rank sum test; comparisons with adjusted BH *P*-values <.05 are shown); the LC-MS peak area of each feature in the early/peak (left side; circles) vs. late phase (triangles) colored by their predicted NPC pathway; error bars represent SDs, and a circle/triangle represents the mean peak area of each feature.

To complement our genomic prediction of secondary metabolism patterns, we aimed at generating a metabolic landscape of the bacterial communities from Days 10 to 113 using liquid chromatography–tandem mass spectrometry (LC-MS/MS). To ease the analysis of this complex data, feature-based molecular networking (MN) was used, allowing for features with similar fragmentation patterns to be linked and form clusters or molecular families. Inherent in this analysis is the idea that structurally similar metabolites will fragment in similar fashions, allowing for researchers to pull more out of complex data through relating features in a more automated process. From a total of 795 features detected in the metabolome, 45, 36, and 476 features were unique to the early (*n* = 23), peak (*n* = 8), and late (*n* = 50) phases, respectively (Supplementary Fig. S6A). A total of 34.36% of the metabolomic feature variance could be described by the first and second components of the PCoA ordination (Fig. 4A). Due to higher beta-dispersion between samples in the late phase compared to the early phase (*P*-value = .002), the significant effect described by the successional phases on metabolome composition (PERMANOVA; R2 = 0.062, *P*-value = .002) may be partly attributed to group heterogeneity as well.

Differential analysis revealed that 14 features, including one predicted polyketide (*m/z* = 300.2169), dominated the early/peak phase compared to 11 features, including one predicted NRP (*m/z* = 494.3458) in a molecular family with two predicted alkaloids (*m/z* = 468.33055, *m/z* = 496.3629), dominating the late phase (Fig. 4B).

Based on the AD amplicon–predicted alpha diversity, we hypothesized that we would find more putative amino acids and peptides during the early/peak phase compared to the late phase. However, the metabolic richness of the putative tripeptides or larger peptides (*m/z* > 300) were similar across all timepoints (Dunnett’s test BH: *P*-value >.05; Supplementary Fig. S6B) and was not comparable with the amplicon-predicted NRPS richness. Thus, the predicted genetic NRPS potential was either not produced, below detection level [37] or further metabolized [8, 38].

Although many microbial secondary metabolites, like nonribosomal peptides, are categorized as having >300 *m/z* [39], low-molecular-weight metabolites, such as tropodithietic acid (*m/z* = 212.9683) [40], have also been described [41]. Consequently, we used a targeted approach to search the molecular network for Mw < 300 *m/z*. One molecular family, a group of quinoline alkaloids (NPC superclass–predicted), was specifically detected at Day 29 in a single replicate (Supplementary Fig. S6C). The GNPS spectral library found matches for three nodes, all pertaining to 2-alkyl-4-quinolones, previously isolated from *Pseudomonas aeruginosa* [42] and *Pseudoalteromonas* spp. [43-45], and known to display various biological activities, including quorum sensing [43, 46]. Multiple known pseudanes (VII, VIII, IX, X, and XI) were detected at the peak phase (Supplementary Fig. S6C), and additional seven new pseudanes (not described to our knowledge) were detected (Supplementary Tab. 2) (Supplementary Fig. S7).

## Discussion

Microbial secondary metabolites are expected to be important biotic modulators of microbial community populations, especially in spatially structured environments [6-8]. Despite the acknowledged importance of secondary metabolites in community interactions, little is known about their dynamics and link to microbial community intrinsic processes. Previous studies have described the microbial community succession of marine biofilm [2, 3], but have not addressed the potential role of secondary metabolites. In other studies, the secondary metabolite potential in a microbial community has been characterized but only at a single time point [14, 15, 18, 47]. Here, we bridge this gap and present the first comprehensive temporal investigation of the interlinkage between secondary metabolite dynamics and metataxonomic composition during community succession in a marine microbial biofilm *in situ*. We show that the microbial secondary metabolite potential changes during marine biofilm succession and that only a few BGC enriched community members drive the BGC succession pattern.

The *in situ* biofilm system was used to assess the general succession pattern for the marine microbial biofilm communities. The early succession (<29 days) revealed increasing numbers of microbial cells (<100 μm) until the arrival of multicellular eukaryotes after Day 29. Chlorophyll *a* concentrations highlighted the prevalence of photosynthetic microorganisms as primary colonizers on the BioElements. This could be explained by the inert surface (plastic) of the BioElements, which does not degrade in the same way as surfaces with organic carbon (e.g. wood). Degradable surfaces typically attract mixotrophs and heterotrophic organisms as primary colonizers, whereas early communities on inert surfaces are primarily composed of autotrophs [3]. Subsequently, the microbial abundance and the eukaryotic richness decreased, which could be attributed to the establishment of sessile bryozoans and an increase in benthic copepoda [48, 49]. Combined, these observations suggest a successional shift around Day 29, which was also observed in the increased variability of chlorophyll *a* densities and divergence of the eukaryotic community compositions after this time point.

Successional shifts have been described in other microbial systems [1-3, 50-52]. They have been suggested to be triggered by both abiotic [53, 54] and biotic factors [52, 55], resulting in “bottom–up” and “top–down” selective pressures [3]. In this study, the successional shift resulted in a significant decrease in the microbial BGC potential, particularly the genomic AD amplicon richness. However, what specific ecological drivers that were causing this succession pattern was not elucidated in our study. It is noteworthy that we observed elevated levels of genes (COG-annotated) encoding stress-related functions at the same time, several of which were related to antimicrobial defense mechanisms [29-36]. Higher levels of stress-related genes can serve as an indicator of a stressful environment [43, 47, 48]; thus, these genes may aid in rapid colonization, as surface colonizers require initial acquisition of space and nutrients. However, it is important to note that the higher levels of stress-related genes and defense-encoding genes identified in this analysis could be attributed to both the secondary metabolite producer itself and non-producing strains. We suggest that secondary metabolite producers in the early succession could contribute to the formation of stressful environments (e.g. increased competition and microbial warfare) or assist in coping with abiotic stress-induced conditions (e.g. nutrient limitations). In contrast, the late succession may favor host-associated microbiomes, which provide a relatively less stressful environment, perhaps because it shields the community from abiotic stress factors [56].

Metagenomic analyses revealed that a high portion of the genomic NRPS potential was concentrated in a few bacterial MAGs, supporting previous findings where it was found that a few taxa were highly enriched with BGCs [15]. These belonged to an unidentified phylum and species within the *Myxococcota* class, which suggests that these organisms may be responsible for inflating the AD potential during the peak phase. Myxobacteria have a huge arsenal of BGCs [57]. They can display complex behaviors such as “pack hunting” predation and fruiting body formation that require a high level of cell-to-cell communication and intercellular coordination mediated by their secondary metabolites [58]. Evidently, microbial secondary metabolism may be vital for the establishment and survival of bacteria in the environment [9, 10, 21, 55], and secondary metabolite producers, found in marine biofilm, may even serve as initiators for the settlement of invertebrates [59]. However, the secondary metabolites may have less impact on the assembly of the invertebrate host–associated dominated microbiomes.

Deciphering the ecological role and function of secondary metabolites has remained a serious challenge, in part due to the *in situ* detection of the metabolites in the environment as well as low structural annotation rates in untargeted metabolomics [18]. Using tandem mass spectrometry, we were able to observe microbial secondary metabolites *in situ* and track changes in the secondary metabolite feature composition during the community succession. The pattern of secondary metabolites was similar in both succession phases, including NRPS-related features, which contrasts with the trend observed in the sequence-based predicted NRPS potential. Furthermore, the low dispersion of the metabolomes indicated by the PCoA suggested a more conserved microbiome during the early succession compared to the late communities. This observation was further supported by the high levels of stress-related genes in the early phase.

Despite methodological advances, very few metabolites can be detected in the marine environment, and even fewer can be annotated [60]. Despite this, we could detect a suite of pseudanes at the successional shift point (Day 29). Pseudanes have multiple biological functions and interactions, including quorum sensing in *Pseudomonas* [61], antimicrobial activity [45], and acting as an interkingdom signal between *Pseudoalteromonas* sp. and microalgae [62]. The presence of Pseudane IV (also named HHQ) has previously been suggested to be structuring marine microbial communities [6, 62]. Since we observe Pseudane IV in the marine community, it is plausible that they contribute to community structuring of both prokaryotes and eukaryotes [62].

Although single pseudane compounds have previously been detected in the environment, such as in tunicate extracts (see Supplementary Table S3), our findings present the first evidence of multiple pseudanes being simultaneously detected *in situ*. However, it is noteworthy that the pseudanes were solely detected in one of the nine replicates, and only at one time point, which could indicate either limitations in chemical detection, or that microbial secondary metabolite production might only occur within a brief time window. Nonetheless, these findings highlight the importance of spatiotemporal resolution when studying microbial assembly dynamics and the secondary metabolite production during biofilm succession.

Our results support similar studies, in that our present metabolite annotation tools are limited when investigating environmental samples, especially from the marine environment [63]. Furthermore, bulk chemical extractions may miss low concentration metabolites, which nevertheless could be important for the microbial community dynamics [64]. Yet, the addition of metabolomes in microbial community ecology is needed to get a comprehensive and holistic perspective on essential factors governing microbial communities. We therefore argue that future studies should aim to implement metabolomes to strengthen the analysis and enhance the cheminformatic tools.

## Conclusion and perspectives

This study has demonstrated that the microbial secondary metabolite potential in marine biofilm communities undergoes succession driven by only a few community members that carry a high genomic potential. This signifies that integration of secondary metabolite investigations into the field of microbial community ecology can aid to a better understanding of intrinsic community processes, such as community succession. Further work should consider a higher spatiotemporal resolution to elucidate the local and temporal effects specifically related to secondary metabolites.

## Materials and methods

### Experimental design, immersion site, and environmental parameters

RK BioElements light (0.93 g/cm^3^; RK Bioelements, Skive, Denmark) were used as solid surface elements to study the biofilm development. In total, 350 BioElements were added to each of the nine (biological replicates, *n* = 9) 0.5-L Nalgene® bottles perforated with multiple holes (diameter ~1 cm) to allow an exchange of water, thus functioned as cages enclosing the BioElements. The cages were immersed in Jyllinge Harbour, Denmark (55.744920 N, 12.094893 E) in June 2021. The attached weights (100 g) allowed the immersion of the cages at 10–20-cm depth. BioElements from cages were sampled on days 4, 7, 14, 28, 42, 56, 70, 84, 98, 112, and 126. Salinity was measured on each sampling day, and water temperatures were measured continuously using a HOBO Pendant® MX Temperature/Light data logger (Onset Computer Corporation, Pocasset, MA).

### Microbial community abundances and chlorophyll *a* levels

At each sampling time point, two BioElements from each biological replicate (*n* = 2 × 9) were removed to estimate microbial community abundances by flow cytometry (Supplementary section 01.01) and to estimate the chlorophyll-*a* concentration (see Supplementary Section 01.02).

### DNA extraction

At each sampling point, a third BioElement was removed from each biological replicate (*n* = 9) in parallel with seawater samples and used for DNA extraction (see Supplementary Section 01.03).

### Amplicon sequencing of the 16S rRNA, 18S rRNA, and adenylation domain gene regions

The 16S rRNA V3–V4 region, 18S rRNA V9 region, and NRP ADs were amplified by the primer pairs tagged with octameric barcodes, cleaned, and pooled in equimolar ratios as previously described [25]. Primer information is listed in Supplementary Table S1. The amplification was carried out in 75-μl reactions. Further information for each region has been specified in the supplementary information (see Supplementary Section 01.04). All amplicons were cleaned and pooled in equimolar ratios. All sequencing was done by Novogene, Cambridge (UK), on the Illumina NovaSeq 6000 250PE (16S V3–V4 and AD amplicons) or 150PE (18S V9) platform.

### Preprocessing and analysis of 16S, 18S, and adenylation domain amplicon sequences

Raw reads were reoriented, and primer sequences were removed from paired-end reads using *Cutadapt* (v.3.7). Reads were processed in *R* (v4.2.2) using *DADA2* (v1.12.1) [65]. First, reads were truncated according to their quality profiles and filtered using the parameters maxN = 0, maxEE = c(2,2) and TrunQ = 2. Error rates were characterized using the first 10^9^ bases and dereplicated. Paired-end 16S V3V4 and 18S V9 reads were merged, and only the forward reads from the AD sequencing were further processed. Sequencing runs were analyzed independently to obtain run-specific error models and combined after read merging. Chimeras were removed, and 16S and 18S ASVs were taxonomically classified based on the *SILVA database* (v.138.1) using the *DADA2*. Contaminants were removed using the prevalence method (prevalence = 0.5) with *decontam* (v. 1.16) [66].

ASVs with <10 reads and 16S ASVs with a length below 380 bp were filtered out. Phylogenetic trees were constructed by aligning sequences with *Mafft* (v. 7.490) [67], using a gap penalty of one, and adjusting sequence direction if necessary. *FastTree* created an approximately maximum-likelihood phylogenetic tree using generalized time-reversible [68]. The tree was rooted by automatically picking an outgroup with the longest branch. AD ASVs were clustered into OBUs (99%) using the function *TreeLine* (method = complete, cutoff = 0.01) from *DECIPHER* (v2.26.0) [69].

### Bacterial amplicon profiling and compositional analysis

A linear mixed-effects (LMEs) model of the relationship between time and environment (fixed effects) on the alpha diversities was conducted using *lme4* (v4_1.1-29) [70], with biological replicates as the random effect. *P*-values were obtained by the *Anova* function from *car* (v1.0-9) and post hoc multiple pairwise comparisons using *emmeans* (EMMs) (v1.7.5) [71], with the inclusion of *P*-value adjustment (Bonferroni). ASV tables were normalized using total sum scaling (TSS) to 100 000 reads per sample for compositional analysis, using Bray–Curtis dissimilarities. Nonmetric dimensional scaling (nMDS) ordination plots were used to visualize the results. The significance of terms was determined by a PERMANOVA test using *adonis2* and *betadisper* from *vegan* (v2.6-2) [72]. *ANCOM-BC* (v1.6.2) [73], with default settings, was used to identify 16S ASVs that were significantly different between Days 23–29 and Days 44–57.

### Metagenomic sequencing

A subset of the samples was used for shotgun sequencing. The extracted DNA was shipped to Novogene (Cambridge, UK) postextraction for DNA fragmentation by sonication. Library preparation was carried out using Novogene NGS DNA Library Prep Set (Cat No. PT004). Further quality control included qPCR for quantification. Size distribution was detected using Bioanalyzer 2100 (Agilent), and quantified libraries were pooled and sequenced on a NovaSeq 6000 (Illumina) with a 150-bp paired-end strategy.

### Genome-resolved metagenomics

Data processing was adopted from Rasmussen *et al.* [74]. In brief, reads were quality-controlled, using *FastQC* (v0.11.8). Removal of adapters and low-quality reads was done with *AdapterRemoval* (v2.3.3) [75]. Duplicates were removed, and reads were repaired to remove singletons using *bbmap* (v.38.35). To increase assembly efficiency by reducing eukaryotic contaminants, data were filtered for the bryozoan (GCA_914767715.1) genome and human (HG19) genome using *minimap2* (v2.6) [76]. Filtered data were coassembled using *MegaHIT* (v1.2.9) with a minimal length of 1000 bp per scaffold, using the meta-large flag for large and complex metagenomes. We used the *anvi’o* pipeline [77] for binning and curating the metagenome, as described in Rasmussen *et al.* [78]. *Anvi’o* was used to profile the scaffolds and identify ORF using *Prodigal* (v2.6.3) [79] and *HMMER* (v.3.355) matching archaeal, bacterial [80], Protista (http://merenlab.org/delmont-euk-scgs) SCG collections, and the universal marker for RNA polymerase A and B [77]. Completeness and redundancy of MAGs were calculated based on SCGs in *anvi’o* databases. MAGs with completeness >50% and <10% redundancy were further processed. Predicted gene functions were annotated using *Pfam* [78] and *COG* [77]. BGCs and functional annotation were predicted by *antiSMASH* (v6.0) [81] and *BiGSCAPE* [82].

### Metabolome extraction and data acquisition

A BioElement from each biological replicate was used for MeOH extraction. Samples were sonicated in a formic acid (0.1%) MeOH solution for 4 min at 25°C (28-kHz, 2 × 150 W sonication bath, Delta 220, Deltasonic, Meaux, France). Subsequently, samples were vortexed at max speed for 30 s, evaporated, eluted in 100 μl MeOH, and analyzed on an LC–MS system. LC was performed on an Agilent Infinity 1290 UHPLC system, where 20-μl extract was injected onto an Agilent Poroshell 120 phenyl-C6column (2.1 × 150 mm, 1.9 μm) at 40°C using CH3CN and H2O, both containing 20 mM formic acid. Initially, a linear gradient of 10% CH3CN/H2O to 100% CH3CN over 10 min was employed, followed by isocratic elution of 100% CH3CN for 2 min. Then, the gradient was returned to 10% CH3CN/H2O in 0.1 min and, finally, an isocratic condition of 10% CH3CN/H2O for 1.9 min, all at a flow rate of 0.35 min/ml. HRMS data were recorded in positive ionization on an Agilent 6545 QTOF MS equipped with an Agilent Dual Jet Stream electrospray ion source with a drying gas temperature of 250°C, drying gas flow of 8 min/L, sheath gas temperature of 300°C, and sheath gas flow of 12 min/L. The capillary voltage was 4000 V, and the nozzle voltage was 500 V. The HRMS data were processed and analyzed using Agilent MassHunter Qualitative Analysis B.07.00. HPLC-grade and LC-MS-grade solvents (VWR Chemicals) were used for extractions and LC-MS, respectively.

### Metabolomic annotation and metabolite substructural analysis

Spectral data were converted to mzML using MSConvert (ProteoWizard) and preprocessed using MZmine 3 [82]. MN was all completed within the *GNPS* platform [83], which includes feature-based MN [84] and ion identity MN [85]. The ion identity MN workflow can be found here: UCSD Computational Mass Spectrometry Website. Formula predictions, compound class annotations, and NPC annotations were run through SIRIUS/v5.5.7 and include SIRIUS [86], ZODIAC [87], CSI:FingerID, and CANOPUS. Visualization of the molecular networks was completed using Cytoscape 3.8.2. [88].

### Metabolomic analysis

For the alpha- and beta-analysis, features with *m/z* < 300 [79], carbohydrates, fatty acids (NPC pathway predicted), and lignans (NPC superclass predicted) were removed to limit metabolome to potential secondary metabolite production. The data were centered log-transformed after zero-handling with zCompositions pseudo-counts as described in Bates *et al*. [89] between the early/peak phase (grouped), and the late phase was carried out using the Kruskal–Wallis rank sum test on the non-transformed LC-MC “peak area” across the samples.

### Overall information for statistical analysis

All analyses were performed using *R* (v4.2.2) in *RStudio*, using *tidyverse* (v1.3.1) [90] for data handling and visualization. Differential abundance analysis of metagenomic genes was done using *DESeq2* (v1.36.0) [91]. Alpha-diversity of bacterial amplicons, AD OBUs, and potential NRPs features (predicted as amino acid and peptides) was calculated as the total observed features per day using the *estimate_richness* from phyloseq (v1.40.0) [92].

## Supplementary Material

clean_SupplementaryInformation_Bech_et_al_15012024y_cae006

Supplementary_Table_2_ycae006

## Data Availability

Sequencing data are available in the Sequencing Read Archive (SRA); 16S, 18S, and AD amplicon data and shotgun sequenced metagenomes in BioProject PRJNA928313. LC-MS2 data are available in MassIVE with accession number MSV000090335. R and shell codes used in this study are available at https://github.com/PKBech/PRJNA928313.

## References

[1] Enke TN , Datta MS, Schwartzman J et al. Modular assembly of polysaccharide-degrading marine microbial communities. Curr Biol 2019;29:1528–1535.e6. 10.1016/j.cub.2019.03.047. 31031118

[2] Datta MS , Sliwerska E, Gore J et al. Microbial interactions lead to rapid micro-scale successions on model marine particles. Nat Commun 2016;7:11965. 10.1038/ncomms11965. 27311813 PMC4915023

[3] Tobias-Hünefeldt SP , Wenley J, Baltar F et al. Ecological drivers switch from bottom–up to top–down during model microbial community successions. ISME J 2021;15:1085–97. 10.1038/s41396-020-00833-6. 33230267 PMC8115227

[4] Fu H , Uchimiya M, Gore J et al. Ecological drivers of bacterial community assembly in synthetic phycospheres. Proc Natl Acad Sci U S A 2020;117:3656–62. 10.1073/pnas.1917265117. 32015111 PMC7035482

[5] Antwis RE , Griffiths SM, Harrison XA et al. Fifty important research questions in microbial ecology. FEMS Microbiol Ecol 2017;93:fix044. 10.1093/femsec/fix044. 28379446

[6] Junkins EN , McWhirter JB, McCall L-I et al. Environmental structure impacts microbial composition and secondary metabolism. ISME Comm 2022;2:2. 10.1038/s43705-022-00097-5. PMC9723690 37938679

[7] Dahlstrom KM , McRose DL, Newman DK. Keystone metabolites of crop rhizosphere microbiomes. Curr Biol 2020;30:R1131–7. 10.1016/j.cub.2020.08.005. 33022255 PMC8059773

[8] Hansen ML , Wibowo M, Jarmusch SA et al. Sequential interspecies interactions affect production of antimicrobial secondary metabolites in *Pseudomonas protegens* DTU9.1. ISME J 2022;16:2680–90. 10.1038/s41396-022-01322-8. 36123523 PMC9666462

[9] D’Onofrio A , Crawford JM, Stewart EJ et al. Siderophores from neighboring organisms promote the growth of uncultured bacteria. Chem Biol 2010;17:254–64. 10.1016/j.chembiol.2010.02.010. 20338517 PMC2895992

[10] Abrudan MI , Smakman F, Grimbergen AJ et al. Socially mediated induction and suppression of antibiosis during bacterial coexistence. Proc Natl Acad Sci U S A 2015;112:11054–9. 10.1073/pnas.1504076112. 26216986 PMC4568218

[11] Yamanaka K , Oikawa H, Ogawa H-O et al. Desferrioxamine E produced by *Streptomyces griseus* stimulates growth and development of *Streptomyces tanashiensis*. Microbiology 2005;151:2899–905. 10.1099/mic.0.28139-0. 16151202

[12] Patin NV , Schorn M, Aguinaldo K et al. Effects of actinomycete secondary metabolites on sediment microbial communities. Appl Environ Microbiol 2017;83:02676–16. 10.1128/AEM.02676-16. PMC5288833 27986719

[13] Zoccarato L , Sher D, Miki T et al. A comparative whole-genome approach identifies bacterial traits for marine microbial interactions. Commun Biol 2022;5:276. 10.1038/s42003-022-03184-4. 35347228 PMC8960797

[14] Charlop-Powers Z , Pregitzer CC, Lemetre C et al. Urban park soil microbiomes are a rich reservoir of natural product biosynthetic diversity. Proc Natl Acad Sci U S A 2016;113:14811–6. 10.1073/pnas.1615581113. 27911822 PMC5187742

[15] Paoli L , Ruscheweyh H-J, Forneris CC et al. Biosynthetic potential of the global ocean microbiome. Nature 2022;607:111–8. 10.1038/s41586-022-04862-3. 35732736 PMC9259500

[16] Borsetto C , Amos GCA, da Rocha UN et al. Microbial community drivers of PK/NRP gene diversity in selected global soils. Microbiome 2019;7:78. 10.1186/s40168-019-0692-8. 31118083 PMC6532259

[17] Cimermancic P , Medema MH, Claesen J et al. Insights into secondary metabolism from a global analysis of prokaryotic biosynthetic gene clusters. Cell 2014;158:412–21. 10.1016/j.cell.2014.06.034. 25036635 PMC4123684

[18] Shaffer JP , Nothias L-F, Thompson LR et al. Standardized multi-omics of Earth’s microbiomes reveals microbial and metabolite diversity. Nat Microbiol 2022;7:2128–50. 10.1038/s41564-022-01266-x. 36443458 PMC9712116

[19] Chung HC , Lee OO, Huang Y-L et al. Bacterial community succession and chemical profiles of subtidal biofilms in relation to larval settlement of the polychaete *Hydroides* elegans. ISME J 2010;4:817–28. 10.1038/ismej.2009.157. 20090788

[20] Zhang W , Ding W, Li Y-X et al. Marine biofilms constitute a bank of hidden microbial diversity and functional potential. Nat Commun 2019;10:517. 10.1038/s41467-019-08463-z. 30705275 PMC6355793

[21] Tan CH , Koh KS, Xie C et al. Community quorum sensing signalling and quenching: microbial granular biofilm assembly. NPJ Biofilms Microbiomes 2015;1:15006. 10.1038/npjbiofilms.2015.6. 28721230 PMC5515215

[22] Nemergut DR , Schmidt SK, Fukami T et al. Patterns and processes of microbial community assembly. Microbiol Mol Biol Rev 2013;77:342–56. 10.1128/MMBR.00051-12. 24006468 PMC3811611

[23] Caruso T , Chan Y, Lacap DC et al. Stochastic and deterministic processes interact in the assembly of desert microbial communities on a global scale. ISME J 2011;5:1406–13. 10.1038/ismej.2011.21. 21368908 PMC3160679

[24] Johansen R , Albright M, Gallegos-Graves LV et al. Tracking replicate divergence in microbial community composition and function in experimental microcosms. Microb Ecol 2019;78:1035–9. 10.1007/s00248-019-01368-w. 30941446

[25] Bech PK , Lysdal KL, Gram L et al. Marine sediments hold an untapped potential for novel taxonomic and bioactive bacterial diversity. mSystems 2020;5:e00782–20. 10.1128/mSystems.00782-20. 32934119 PMC7498687

[26] Amiri Moghaddam J , Crüsemann M, Alanjary M et al. Analysis of the genome and metabolome of marine myxobacteria reveals high potential for biosynthesis of novel specialized metabolites. Sci Rep 2018;8:16600. 10.1038/s41598-018-34954-y. 30413766 PMC6226438

[27] Belknap KC , Park CJ, Barth BM et al. Genome mining of biosynthetic and chemotherapeutic gene clusters in Streptomyces bacteria. Sci Rep 2020;10:2003. 10.1038/s41598-020-58904-9. 32029878 PMC7005152

[28] Rausch C , Weber T, Kohlbacher O et al. Specificity prediction of adenylation domains in nonribosomal peptide synthetases (NRPS) using transductive support vector machines (TSVMs). Nucleic Acids Res 2005;33:5799–808. 10.1093/nar/gki885. 16221976 PMC1253831

[29] Chetri S , Bhowmik D, Paul D et al. AcrAB-TolC efflux pump system plays a role in carbapenem non-susceptibility in *Escherichia coli*. BMC Microbiol 2019;19:210. 10.1186/s12866-019-1589-1. 31488061 PMC6727511

[30] Tanabe M , Szakonyi G, Brown KA et al. The multidrug resistance efflux complex, EmrAB from *Escherichia coli* forms a dimer in vitro. Biochem Biophys Res Commun 2009;380:338–42. 10.1016/j.bbrc.2009.01.081. 19171121

[31] Phelps HA , Neely MN. SalY of the *Streptococcus pyogenes* lantibiotic locus is required for full virulence and intracellular survival in macrophages. Infect Immun 2007;75:4541–51. 10.1128/IAI.00518-07. 17576754 PMC1951192

[32] Newton GL , Leung SS, Wakabayashi JI et al. The DinB superfamily includes novel mycothiol, bacillithiol, and glutathione S-transferases. Biochemistry 2011;50:10751–60. 10.1021/bi201460j. 22059487 PMC3232311

[33] Francis JW , Royer CJ, Cook PD. Structure and function of the bacillithiol-S-transferase BstA from *Staphylococcus aureus*. Protein Sci 2018;27:898–902. 10.1002/pro.3384. 29417696 PMC5866932

[34] Tyagi R , Eswaramoorthy S, Burley SK et al. A common catalytic mechanism for proteins of the HutI family. Biochemistry 2008;47:5608–15. 10.1021/bi800180g. 18442260 PMC3232013

[35] Griggs DW , Konisky J. Mechanism for iron-regulated transcription of the *Escherichia coli* cir gene: metal-dependent binding of fur protein to the promoters. J Bacteriol 1989;171:1048–54. 10.1128/jb.171.2.1048-1054.1989. 2644221 PMC209700

[36] Kvint K , Nachin L, Diez A et al. The bacterial universal stress protein: function and regulation. Curr Opin Microbiol 2003;6:140–5. 10.1016/S1369-5274(03)00025-0. 12732303

[37] Buijs Y , Isbrandt T, Zhang S-D et al. The antibiotic andrimid produced by *Vibrio coralliilyticus* increases expression of biosynthetic gene clusters and antibiotic production in *Photobacterium galatheae*. Front Microbiol 2020;11:1–13. 10.3389/fmicb.2020.622055. PMC7793655 33424823

[38] Buijs Y , Zhang S-D, Jørgensen KM et al. Enhancement of antibiotic production by co-cultivation of two antibiotic producing marine *Vibrionaceae strains*. FEMS Microbiol Ecol 2021;97:fiab041. 10.1093/femsec/fiab041. 33693627

[39] Newman DJ , Cragg GM. Natural products as sources of new drugs over the nearly four decades from 01/1981 to 09/2019. J Nat Prod 2020;83:770–803. 10.1021/acs.jnatprod.9b01285. 32162523

[40] Brinkhoff T , Bach G, Heidorn T et al. Antibiotic production by a Roseobacter clade-affiliated species from the German Wadden Sea and its antagonistic effects on indigenous isolates. Appl Environ Microbiol 2004;70:2560–5. 10.1128/AEM.70.4.2560-2565.2003. 15066861 PMC383154

[41] van Santen JA , Poynton EF, Iskakova D et al. The Natural Products Atlas 2.0: a database of microbially-derived natural products. Nucleic Acids Res 2021;50:D1317–23. 10.1093/nar/gkab941. PMC8728154 34718710

[42] Li J , Sun W, Saalim M et al. Isolation of 2-alkyl-4-quinolones with unusual side chains from a Chinese isolate. J Nat Prod 2020;83:2294–8. 10.1021/acs.jnatprod.0c00026. 32603106

[43] Harvey EL , Deering RW, Rowley DC et al. A bacterial quorum-sensing precursor induces mortality in the marine coccolithophore, *Emiliania huxleyi*. Front Microbiol 2016;7:59. 10.3389/fmicb.2016.00059. 26870019 PMC4737879

[44] Kim WJ , Kim YO, Kim JH et al. Liquid chromatography-mass spectrometry-based rapid secondary-metabolite profiling of marine *Pseudoalteromonas* sp. M2. Mar Drugs 2016;14:24. 10.3390/md14010024. 26805856 PMC4728520

[45] Paulsen SS , Isbrandt T, Kirkegaard M et al. Production of the antimicrobial compound tetrabromopyrrole and the Pseudomonas quinolone system precursor, 2-heptyl-4-quinolone, by a novel marine species *Pseudoalteromonas galatheae* sp. nov. Sci Rep 2020;10:21630. 10.1038/s41598-020-78439-3. 33303891 PMC7730127

[46] Déziel E , Lépine F, Milot S et al. Analysis of *Pseudomonas aeruginosa* 4-hydroxy-2-alkylquinolines (HAQs) reveals a role for 4-hydroxy-2-heptylquinoline in cell-to-cell communication. Proc Natl Acad Sci U S A 2004;101:1339–44. 10.1073/pnas.0307694100. 14739337 PMC337054

[47] Gavriilidou A , Kautsar SA, Zaburannyi N et al. Compendium of specialized metabolite biosynthetic diversity encoded in bacterial genomes. Nat Microbiol 2022;7:726–35. 10.1038/s41564-022-01110-2. 35505244

[48] Dhont J , Dierckens K, Støttrup J et al. Rotifers, Artemia and copepods as live feeds for fish larvae in aquaculture. Adv Aquacul Hatchery Technol 2013;242:157–202. 10.1533/9780857097460.1.157.

[49] Dahms H-U , Dobretsov S, Qian P-Y. The effect of bacterial and diatom biofilms on the settlement of the bryozoan Bugula neritina. J Exp Mar Biol and Ecol 2004;313:191–209. 10.1016/j.jembe.2004.08.005.

[50] Gralka M , Szabo R, Stocker R et al. Trophic interactions and the drivers of microbial community assembly. Curr Biol 2020;30:R1176–88. 10.1016/j.cub.2020.08.007. 33022263

[51] Briand J-F , Pochon X, Wood SA et al. Metabarcoding and metabolomics offer complementarity in deciphering marine eukaryotic biofouling community shifts. Biofouling 2018;34:657–72. 10.1080/08927014.2018.1480757. 30185057

[52] Gilbert JA , Steele JA, Caporaso JG et al. Defining seasonal marine microbial community dynamics. ISME J 2012;6:298–308. 10.1038/ismej.2011.107. 21850055 PMC3260500

[53] Lucas J , Wichels A, Teeling H et al. Annual dynamics of North Sea bacterioplankton: seasonal variability superimposes short-term variation. FEMS Microbiol Ecol 2015;91:fiv099. 10.1093/femsec/fiv099. 26298013

[54] Bunse C , Pinhassi J. Marine bacterioplankton seasonal succession dynamics. Trends Microbiol 2017;25:494–505. 10.1016/j.tim.2016.12.013. 28108182

[55] Romdhane S , Spor A, Aubert J et al. Unraveling negative biotic interactions determining soil microbial community assembly and functioning. ISME J 2022;16:296–306. 10.1038/s41396-021-01076-9. 34321619 PMC8692615

[56] Boscaro V , Holt CC, Van Steenkiste NWL et al. Microbiomes of microscopic marine invertebrates do not reveal signatures of phylosymbiosis. Nat Microbiol 2022;7:810–9. 10.1038/s41564-022-01125-9. 35618773

[57] Loureiro C , Galani A, Gavriilidou A et al. Comparative metagenomic analysis of biosynthetic diversity across sponge microbiomes highlights metabolic novelty, conservation, and diversification. mSystems 2022;7:e0035722. 10.1128/msystems.00357-22. 35862823 PMC9426513

[58] Cao P , Dey A, Vassallo CN et al. How myxobacteria cooperate. J Mol Biol 2015;427:3709–21. 10.1016/j.jmb.2015.07.022. 26254571 PMC4658263

[59] Salta M , Wharton JA, Blache Y et al. Marine biofilms on artificial surfaces: structure and dynamics. Environ Microbiol 2013;15:2879–93. 10.1111/1462-2920.12186. 23869714

[60] Bouslimani A , Sanchez LM, Garg N et al. Mass spectrometry of natural products: current, emerging and future technologies. Nat Prod Rep 2014;31:718–29. 10.1039/c4np00044g. 24801551 PMC4161218

[61] Reen FJ , Mooij MJ, Holcombe LJ et al. The Pseudomonas quinolone signal (PQS), and its precursor HHQ, modulate interspecies and interkingdom behaviour. FEMS Microbiol Ecol 2011;77:413–28. 10.1111/j.1574-6941.2011.01121.x. 21539583

[62] Whalen KE , Becker JW, Schrecengost AM et al. Bacterial alkylquinolone signaling contributes to structuring microbial communities in the ocean. Microbiome 2019;7:93. 10.1186/s40168-019-0711-9. 31208456 PMC6580654

[63] Chase AB , Bogdanov A, Demko AM et al. Biogeographic patterns of biosynthetic potential and specialized metabolites in marine sediments. ISME J 2023;17:976–83. 10.1038/s41396-023-01410-3. 37061583 PMC10284892

[64] Sogin ML , Morrison HG, Huber JA et al. Microbial diversity in the deep sea and the underexplored ‘rare biosphere’. Proc Natl Acad Sci U S A 2006;103:12115–20. 10.1073/pnas.0605127103. 16880384 PMC1524930

[65] Callahan BJ , McMurdie PJ, Rosen MJ et al. DADA2: high-resolution sample inference from Illumina amplicon data. Nat Methods 2016;13:581–3. 10.1038/nmeth.3869. 27214047 PMC4927377

[66] Davis NM , Proctor DM, Holmes SP et al. Simple statistical identification and removal of contaminant sequences in marker-gene and metagenomics data. Microbiome 2018;6:226. 10.1186/s40168-018-0605-2. 30558668 PMC6298009

[67] Katoh K , Standley DM. MAFFT multiple sequence alignment software version 7: improvements in performance and usability. Mol Biol Evol 2013;30:772–80. 10.1093/molbev/mst010. 23329690 PMC3603318

[68] Price MN , Dehal PS, Arkin AP. FastTree: computing large minimum evolution trees with profiles instead of a distance matrix. Mol Biol and Evol 2009;26:1641–50. 10.1093/molbev/msp077. 19377059 PMC2693737

[69] Wright E . Using DECIPHER v2.0 to analyze big biological sequence data in R. R J 2016;8:352. 10.32614/RJ-2016-025.

[70] Shannon P , Markiel A, Ozier O et al. Cytoscape: a software environment for integrated models of biomolecular interaction networks. Genome Res 2003;13:2498–504. 10.1101/gr.1239303. 14597658 PMC403769

[71] Wickham H , Averick M, Bryan J et al. Welcome to the tidyverse. J Open Source Software 2019;4:1686. 10.21105/joss.01686.

[72] Dixon P . VEGAN, a package of R functions for community ecology. J Veg Sci 2003;14:927–30. 10.1111/j.1654-1103.2003.tb02228.x.

[73] McMurdie PJ , Holmes S. phyloseq: An R package for reproducible interactive analysis and graphics of microbiome census data. PLoS One 2013;8:e61217. 10.1371/journal.pone.0061217. 23630581 PMC3632530

[74] Rasmussen JA , Villumsen KR, Duchêne DA et al. Genome-resolved metagenomics suggests a mutualistic relationship between Mycoplasma and salmonid hosts. Commun Biol 2021;4:579. 10.1038/s42003-021-02105-1. 33990699 PMC8121932

[75] Lindgreen S . AdapterRemoval: easy cleaning of next-generation sequencing reads. BMC Res Notes 2012;5:337. 10.1186/1756-0500-5-337. 22748135 PMC3532080

[76] Li H . Minimap and miniasm: fast mapping and de novo assembly for noisy long sequences. Bioinformatics 2016;32:2103–10. 10.1093/bioinformatics/btw152. 27153593 PMC4937194

[77] Eren AM , Murat Eren A, Esen ÖC et al. Anvi’o: an advanced analysis and visualization platform for ‘omics data. PeerJ 2015;3:e1319. 10.7717/peerj.1319. 26500826 PMC4614810

[78] Hyatt D , Chen G-L, LoCascio PF et al. Prodigal: prokaryotic gene recognition and translation initiation site identification. BMC Bioinformatics 2010;11:119. 10.1186/1471-2105-11-119. PMC2848648 20211023

[79] Lee MD . GToTree: a user-friendly workflow for phylogenomics. Bioinformatics 2019;35:4162–4. 10.1093/bioinformatics/btz188. 30865266 PMC6792077

[80] Guglielmini J , Woo AC, Krupovic M et al. Diversification of giant and large eukaryotic dsDNA viruses predated the origin of modern eukaryotes. Proc Natl Acad Sci U S A 2019;116:19585–92. 10.1073/pnas.1912006116. 31506349 PMC6765235

[81] El-Gebali S , Mistry J, Bateman A et al. The Pfam protein families database in 2019. Nucleic Acids Res 2019;47:D427–32. 10.1093/nar/gky995. 30357350 PMC6324024

[82] Tatusov RL , Galperin MY, Natale DA et al. The COG database: a tool for genome-scale analysis of protein functions and evolution. Nucleic Acids Res 2000;28:33–6. 10.1093/nar/28.1.33. 10592175 PMC102395

[83] Blin K , Shaw S, Kloosterman AM et al. antiSMASH 6.0: improving cluster detection and comparison capabilities. Nucl Acids Res 2021;49:W29–35. 10.1093/nar/gkab335. 33978755 PMC8262755

[84] Navarro-Muñoz JC , Selem-Mojica N, Mullowney MW et al. A computational framework to explore large-scale biosynthetic diversity. Nat Chem Biol 2020;16:60–8. 10.1038/s41589-019-0400-9. 31768033 PMC6917865

[85] Pluskal T , Castillo S, Villar-Briones A et al. MZmine 2: modular framework for processing, visualizing, and analyzing mass spectrometry-based molecular profile data. BMC Bioinformatics 2010;11:395. 10.1186/1471-2105-11-395. 20650010 PMC2918584

[86] Wang M , Carver JJ, Phelan VV et al. Sharing and community curation of mass spectrometry data with Global Natural Products Social Molecular Networking. Nat Biotechnol 2016;34:828–37. 10.1038/nbt.3597. 27504778 PMC5321674

[87] Nothias L-F , Petras D, Schmid R et al. Feature-based molecular networking in the GNPS analysis environment. Nat Methods 2020;17:905–8. 10.1038/s41592-020-0933-6. 32839597 PMC7885687

[88] Schmid R , Petras D, Nothias L-F et al. Ion identity molecular networking for mass spectrometry-based metabolomics in the GNPS environment. Nat Commun 2021;12:3832. 10.1038/s41467-021-23953-9. 34158495 PMC8219731

[89] Dührkop K , Fleischauer M, Ludwig M et al. SIRIUS 4: a rapid tool for turning tandem mass spectra into metabolite structure information. Nat Methods 2019;16:299–302. 10.1038/s41592-019-0344-8. 30886413

[90] Ludwig M , Nothias L-F, Dührkop K et al. Database-independent molecular formula annotation using Gibbs sampling through ZODIAC. Nat Mach Intell 2020;2:629–41. 10.1038/s42256-020-00234-6.

[91] Love MI , Huber W, Anders S. Moderated estimation of fold change and dispersion for RNA-seq data with DESeq2. Genome Biol 2014;15:550. 10.1186/s13059-014-0550-8. 25516281 PMC4302049

[92] Bates D , Mächler M, Bolker B et al. Fitting linear mixed-effects models using lme4. J Stat Software 2015;67:67. 10.18637/jss.v067.i01.

