# Supplementary material for: Succession of microbial community composition and secondary metabolism during marine biofilm development
Source: ISME Commun. 2024 Jan 20;4(1):ycae006. doi: 10.1093/ismeco/ycae006 (PMC10881302; doi:10.1093/ismeco/ycae006)
Supplement: clean_SupplementaryInformation_Bech_et_al_15012024y_cae006 [file clean_supplementaryinformation_bech_et_al_15012024y_cae006.docx]

Supplementary Information

**Succession of microbial community composition and secondary metabolism during marine biofilm development**

###

Pernille Kjersgaard Bech^1^*, Scott A. Jarmusch^1^, Jacob Agerbo Rasmussen^2^, Morten Tønsberg Limborg^2^, Lone Gram^1^, Nathalie N. Suhr Eiris Henriksen^1^*

* These authors contributed equally

# Corresponding authors: Nathalie N. Suhr Eiris Henriksen, Department of Biotechnology and Biomedicine, Technical University of Denmark, Søltofts Plads, Bldg. 221, DK-2800 Kgs. Lyngby, Denmark, [nasuh@dtu.dk](mailto:nasuh@dtu.dk)

1. Department of Biotechnology and Biomedicine, Technical University of Denmark, Søltofts Plads, Bldg. 221, DK-2800 Kgs. Lyngby, Denmark

2. Center for Evolutionary Hologenomics, Globe Institute, Copenhagen University, Øster Farimagsgade 5, DK.1014 København K, Denmark.

## **01 Supplementary Material & Methods**

### **Supplementary 01.01:** Flow cytometry

At each sampling time point, one BioElement from each biological replicate (*N* = 9) was removed to estimate microbial community abundances by flow cytometry. To remove any organisms that were not firmly attached to the BioElements, each BioElement was dipped in 1.5% Instant Ocean® Sea Salt (IO; Aquarium Systems Inc., Sarrebourg, France) solution. Subsequently, each BioElement was placed in two mL 1.5% IO followed by sonication for 4 min at 25°C (28-kHz, 2 × 150W sonication bath, Delta 220, Deltasonic, Meaux, France). Samples were vortexed at maximum speed for 30 sec and transferred to two mL Eppendorf tubes. Samples were fixed with 55 µL of a 36.7% formaldehyde solution and stored at -80°C until analysis. Microbial abundances (< 100 µm) were estimated using a MACSQuant VYB Flow cytometer (Miltenyi Biotec, North Rhine-Westphalia, Germany). Samples were thawed at room temperature, and 500 µL were filtered using pluriStrainer Mini 100 µm (pluriSelect Life Science, Leipzig, Germany) and stained for 15 min with a 2X SYBR® Green I (Invitrogen™ S7563 by Thermo Fisher Scientific Inc., Eugene, OR, USA). SYBR Green positive cells were detected by the blue laser (488 nm) and filter B1 (525/50 nm). Single events were identified on the SSC-H vs SSC-A plot and gated into the FIT-C vss. SSC-A plot, where SYBR Green positive cells were identified. Data were acquired using the MACSQuantify™ software (Miltenyi Biotec, North Rhine-Westphalia, Germany), and abundance of each group was expressed as cells per BioElement. Sterile BioElements, unstained samples and 1.5% IO stained with 2X SYBR® Green were used as negative controls and to allow adjustment of gates.

### **Supplementary 01.02:** Chlorophyll a levels

At each sampling time point, a second BioElement was removed from each biological replicate (*N* = 9) to estimate the chlorophyll-*a* concentration. After sampling, the BioElements were stored at -80°C until analysis. Two mL of 96% ethanol was added to each BioElement after which they were incubated overnight at room temperature in a dark room, and Chl-*a* measured using a Turner TD-700 fluorometer (Turner designs, San Jose, CA, USA) [[1]](https://paperpile.com/c/WZ4GiO/zTPPO). Sterile BioElements were used as negative controls. The Chl-*a* concentrations were expressed as Chl-*a* µg per BioElement.

### **Supplementary 01.03:** DNA Extraction

Three replicates of 100 mL seawater samples (*N* = 3) were collected and filtered through 0.22 µm Sterivex-GV Durapore® filtration units (Merck, Darmstadt, Germany) and subsequently stored at -80°C.

At each sampling point, a third BioElement was removed from each biological replicate (*N* = 9). For BioElements, microbial cells were removed by sonication and vortexing as described above and transferred to two mL Eppendorf tubes. Samples were centrifuged (8 000 x *g*, 20 min). The pellet was resuspended in 400 µL lysis buffer (400 mM sodium chloride, 750 mM sucrose, 20 mM EDTA, 50 mM Tris-HCl, pH 8.5) and stored at -80°C until DNA extraction.

BioElement and seawater samples were thawed at room temperature. The filters inside the filtration unit of the seawater samples were freed and submerged in a 1.2 mL lysis buffer. Subsequently, BioElements and seawater samples were incubated at 37°C for 30 min with the addition of 1 mg mL^-1^ lysozyme (Sigma, St. Louis, MO, USA), 1% sodium dodecyl sulfate (SDS), and 0.1 mg mL^-1^ proteinase K (Sigma, St. Louis, MO, USA). After incubation overnight at 55°C with regular agitation, samples were centrifuged at 3,000 × g for 5 min. The supernatants were transferred to the lysate chamber in the Maxwell® 16 LEV Blood DNA Kit (Promega, Madison, WI, USA) and processed with the Maxwell™ 16 instrument. Finally, DNA was eluted into a 50 µL TE elution buffer in the Maxwell® 16 LEV Blood DNA Kit. Sterile 0.22 µm Sterivex-GV Durapore® filtration units and BioElements were negative controls.

### **Supplementary 01.04:** Amplicon sequencing of the 16S rRNA, 18S rRNA and AD gene regions.

### The 16S rRNA V3-V4 region was amplified by the primer pairs Fw_V3V4: 5’-CCTACGGGNGGCWGCAG-3’ and Rv_V3V4: 5’-GACTACHVGGGTATCTAATCC-3’ tagged with octameric barcodes, cleaned, and pooled in equimolar ratios as previously described [[2]](https://paperpile.com/c/WZ4GiO/zER5f). The 18S rRNA V9 region (121bp) was amplified with the primer pair 1389_F 5′-TTGTACACACCGCCC-3′ and 1510_R 5′-CCTTCYGCAGGTTCACCTAC-3′ [[2]](https://paperpile.com/c/WZ4GiO/zER5f) tagged with octameric barcodes in 75 μl PCR reaction containing 4 μl template DNA, 28.7 μl DNase-free water, 37.5 μl TEMPase Hot Start 2× Master Mix (VWR International, Søborg, Denmark), 4.8 μl primer pair (10 μM). Amplification conditions for 18S V9 were as follows: 95°C for 15 min, followed by 25 cycles, with 1 cycle consisting of 95°C for 30 s, 57°C for 30 s, and 72°C for 30 s, and a final step of 72°C for 5 min. Degenerate primer pairs targeting NRP adenylation (AD; 750bp) domains A3F0X 5′-GCSTACSYSATSTACACSTCSGG and A7R0X 5′-SASGTCVCCSGTSCGGTA [[2]](https://paperpile.com/c/WZ4GiO/zER5f) were used in 75  μl PCR reaction containing 4 μl template DNA, 23.9 μl DNase-free water, 37.5 μl TEMPase Hot Start 2× Master Mix (VWR International, Søborg, Denmark), 9.6 μl primer pair (10 μM). Amplification conditions for AD domains were as follows: 95°C for 15 min, followed by 40 cycles, with 1 cycle consisting of 95°C for 30 s, 56°C for 30 s, and 72°C for 45 s, and a final step of 72°C for 5 min.

**Supplementary Table S1.** Amplicon Information for 16S, 18S and AD amplicons, including target, regions, length and primer sequences for forward and reverse.

| **Target** | **Region** | **Length (bases)** | **Forward Primer** | **Reverse Primer** |
| --- | --- | --- | --- | --- |
| Bacteria | 16S rRNA V3V4 region | 470 | *Fw_V3V4:* 5’-CCTACGGGNGGCWGCAG-3 | *Rv_V3V4:* 5’-GACTACHVGGGTATCTAATCC-3’ |
| Eukaryotes | 18S rRNA V9 region | 121 | *1389_F:* 5′-TTGTACACACCGCCC-3′ | *1510_R:* 5′-CCTTCYGCAGGTTCACCTAC-3′ |
| NRP adenylation | NRP adenylation (AD) domains | 750 | *A3F0X:* 5′-GCSTACSYSATSTACACSTCSGG | *A7R0X:* 5′-SASGTCVCCSGTSCGGTA |

##

## **02 Supplementary Results**


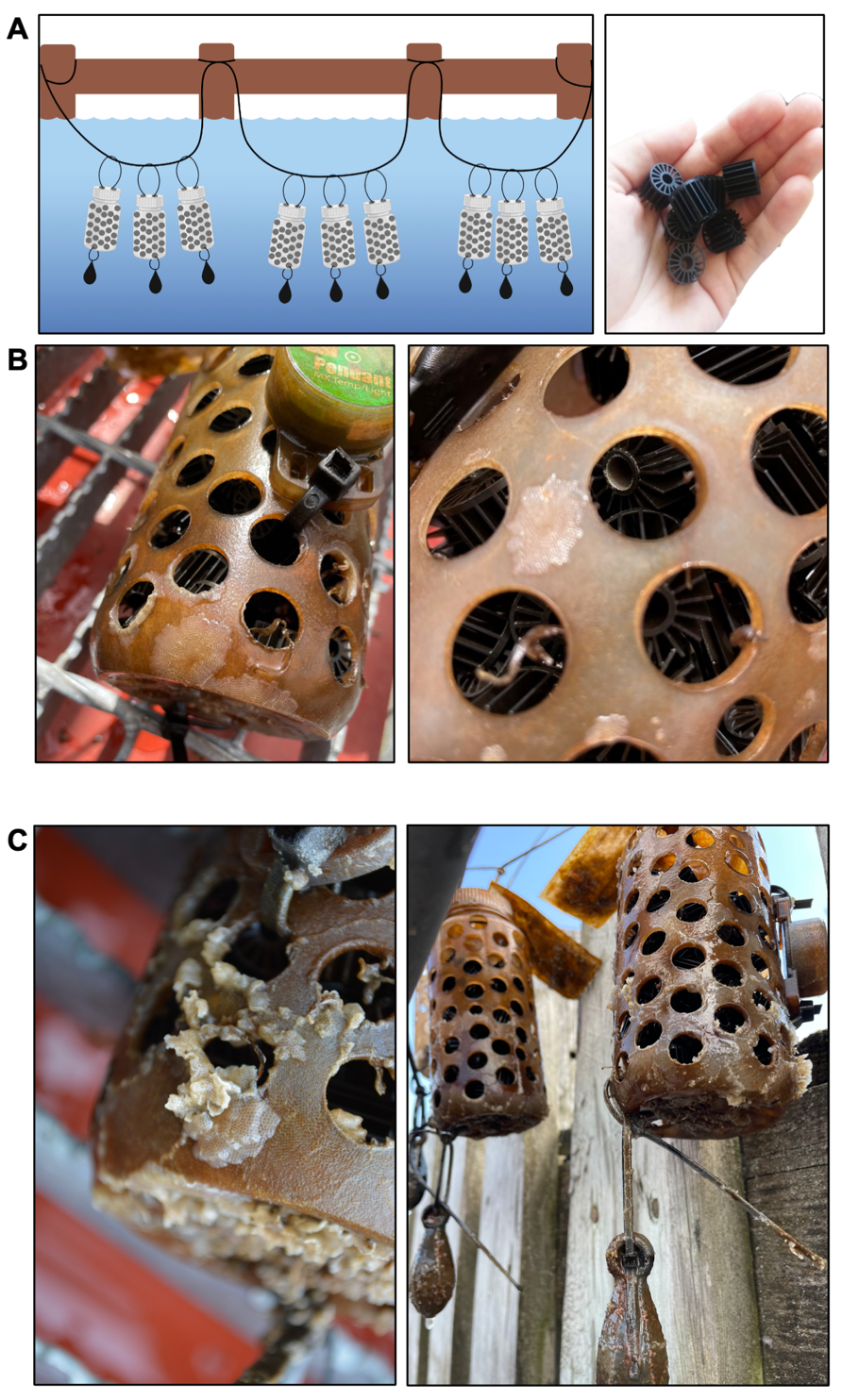


**Supplementary** **Figure 1. Overview of biofilm model system**.

**A)** Left: Illustration of the nine replicates (cages/bottles). Created with Biorender.com. Right: BioElements. **B)** First visual observation of bryozoan (*Conopeum seurati*; visual identification of distinct morphological features) colonization at day 44. Left: colonization on the bottle, right: colonization of a BioElement. **C)** *C. seurati* growth at day 58.

## **
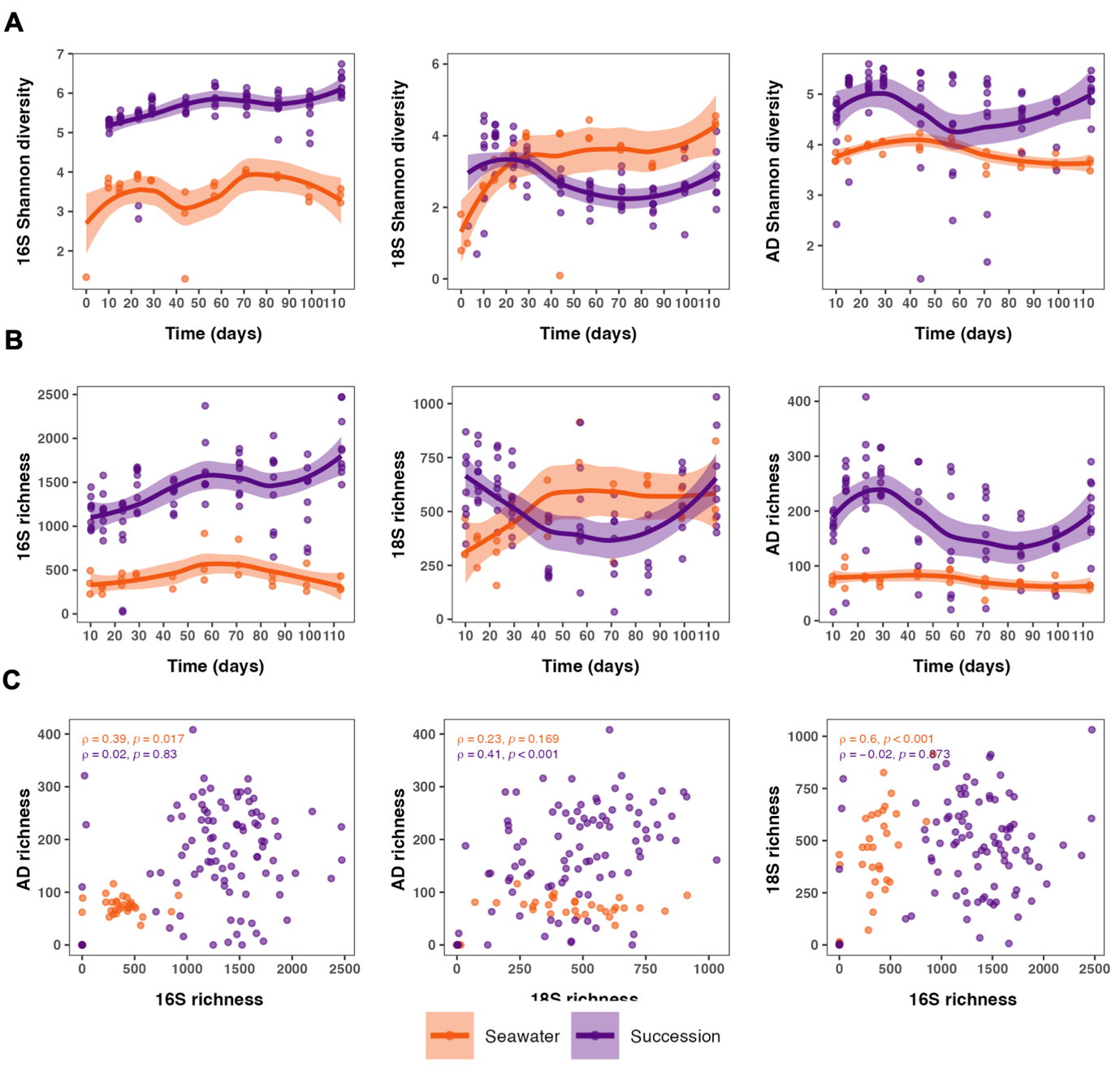
Supplementary Figure 2**. Temporal patterns and correlations of prokaryotic (16S), eukaryotic (18S) and adenylation domains (AD) ASV/OBU diversity in marine biofilm (purple) and seawater (orange). **A)** Shannon diversity over time **B)** Observed richness over time of biofilm and seawater samples. Each point represents one sample. The solid lines represent a loess regression and ribbons the 95% confidence intervals. **C)** Spearman correlations (*N* = 9).

##
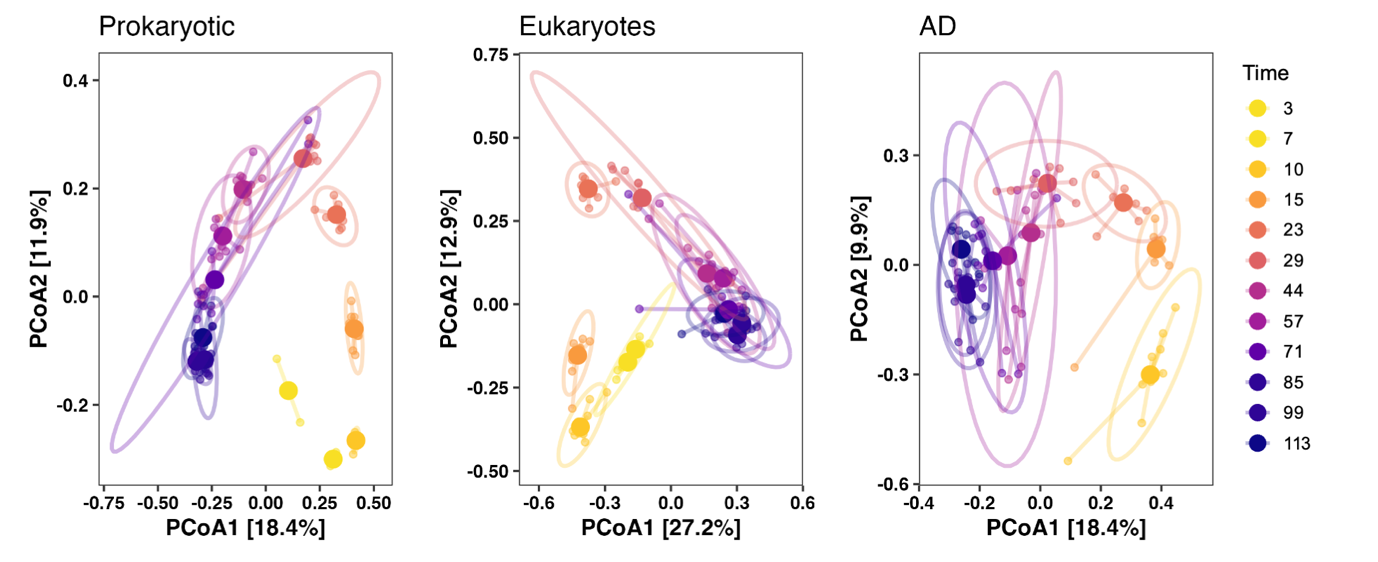


## **Supplementary Figure 3.** Principal Coordinate Analysis (PCoA) of the prokaryotic (16S) and eukaryotic (18S) community compositions over time. Color denotes sampling time point.

## **
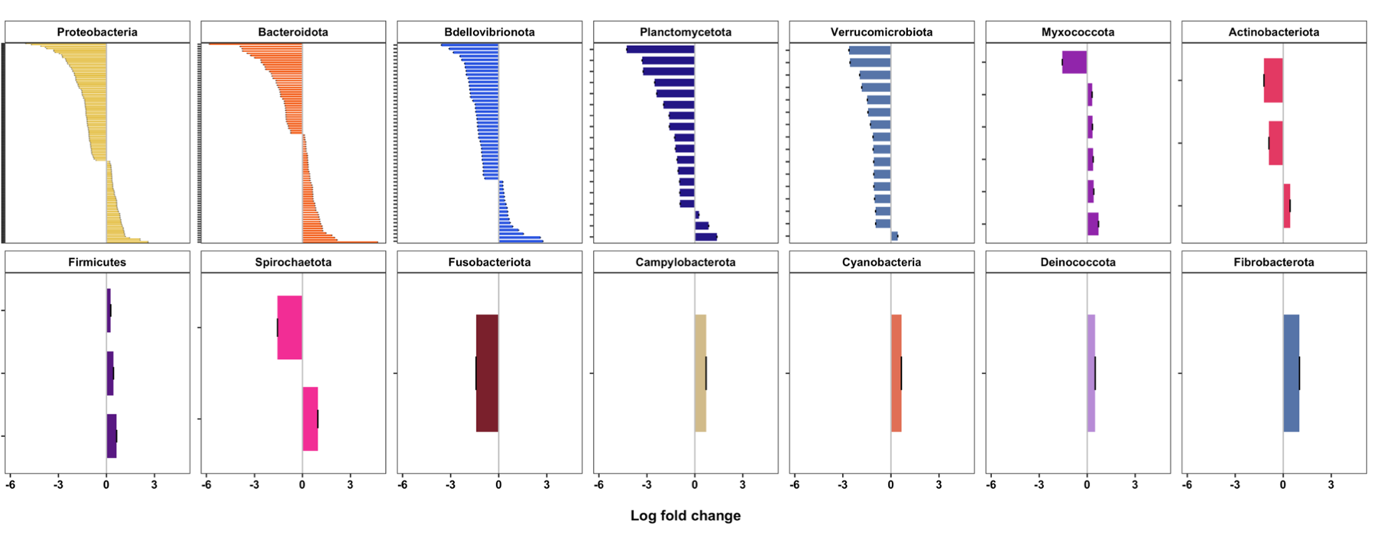
Supplementary Figure 4.** Differential abundances between 16S ASVs. Each bar represent the Log2 fold change of a unique ASV belonging to the specific bacterial phylum between day 23 and 29 (grouped) relative to day 44 and 58 (grouped) calculated by the ANCOM-BC model. For instance, the relative abundance of five ASVs belonging to the Myxococcota phylum have increased at day 44 and 58 (positive log2fold change), and one Myxococcota ASV had a significantly higher relative abundance at day 23 and 29 compared to day 44 and 58 (negative log2fold change). *N* = 9.

##
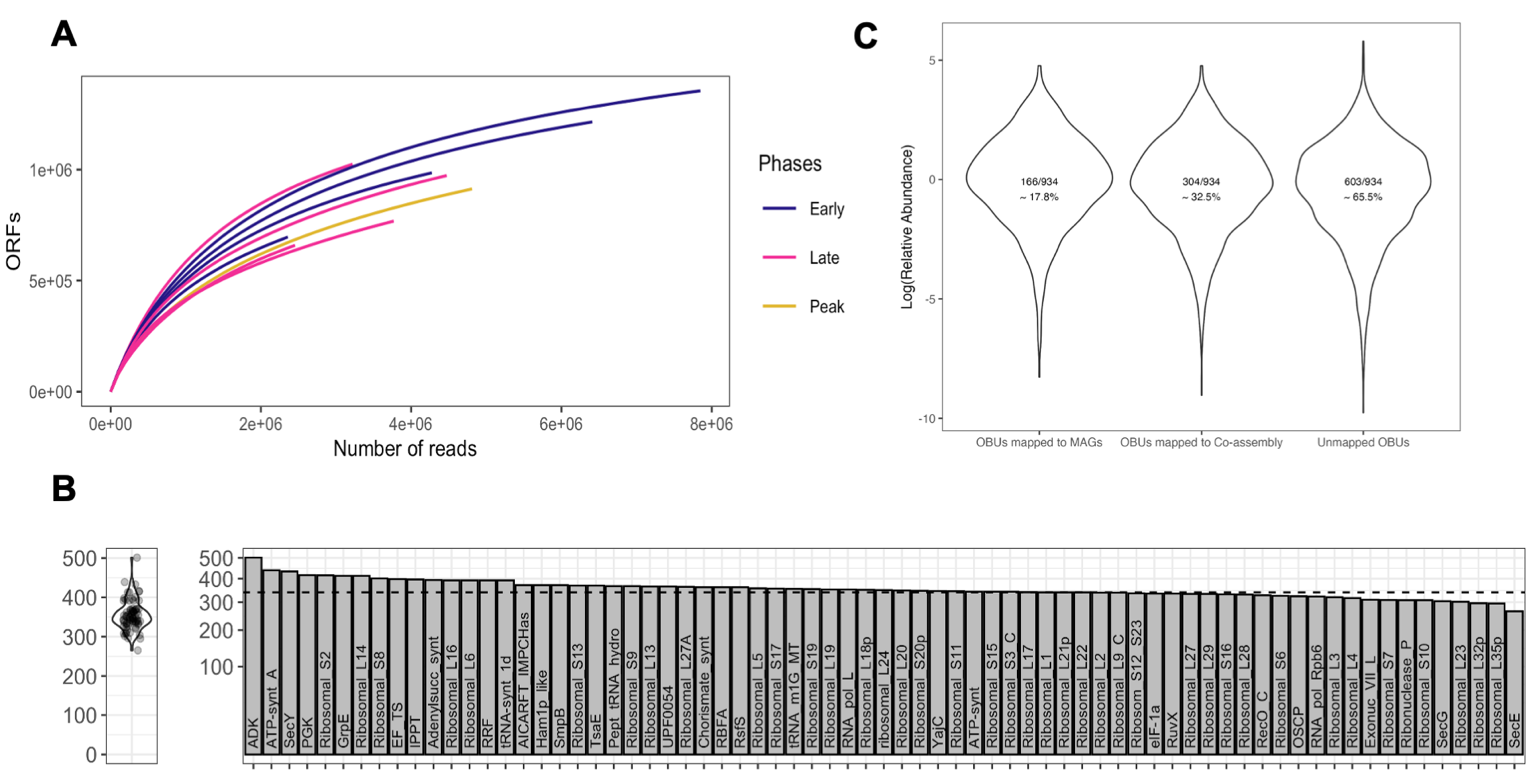


## **Supplementary Figure 5.** Metagenomic variation across time recovered from co-assembled samples. **A)** Rarefaction curves of gene calls (ORFs) found due to sequencing depth. Curves are coloured according to phase (early, peak, and late)**.** *N* = 9**. B)** Density plot and bar plot of bacterial single-copy genes (SCGs) used to estimate the number of genomes in the metagenomes. The dashed line in the bar plot indicates the highest density found of counted bacterial SCGs. **C)** The relationship between all AD OBUs (934) and metagenomes. The respective amount of AD OBUs possible to map back to MAGs (166), the co-assembly (304), and the amount of unmapped AD OBUs (603). The log10(relative abundance) of AD OBUs is given on the y-axis.

| **A**  **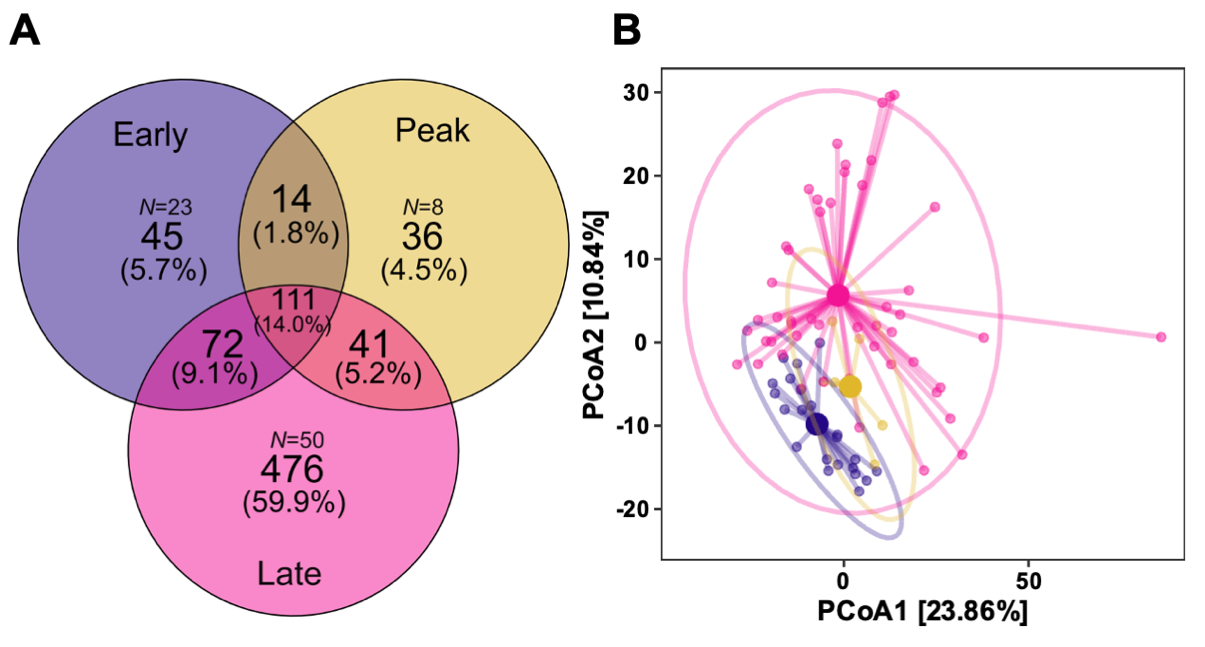** | **B**  **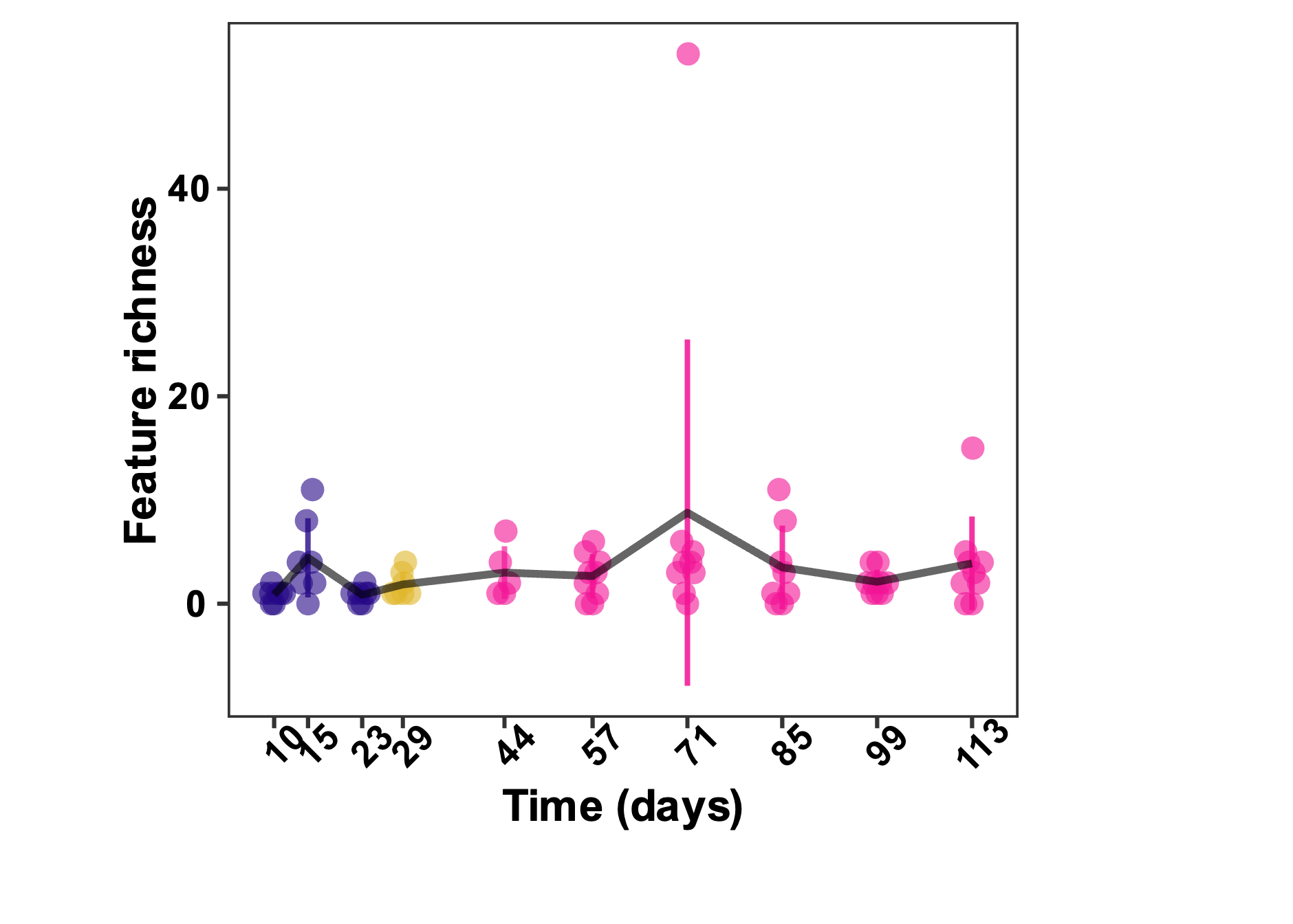 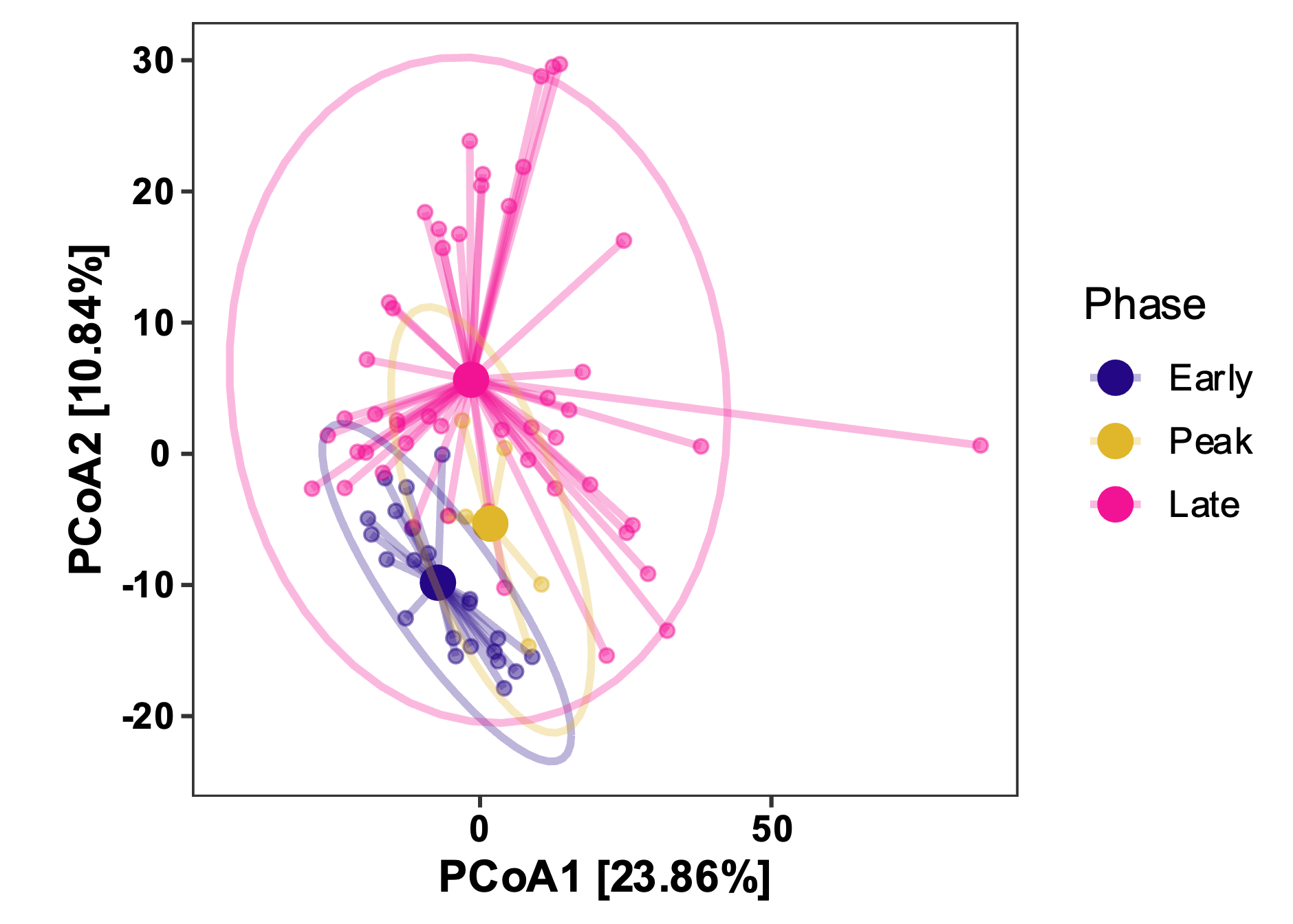** |
| --- | --- |
| **C**  **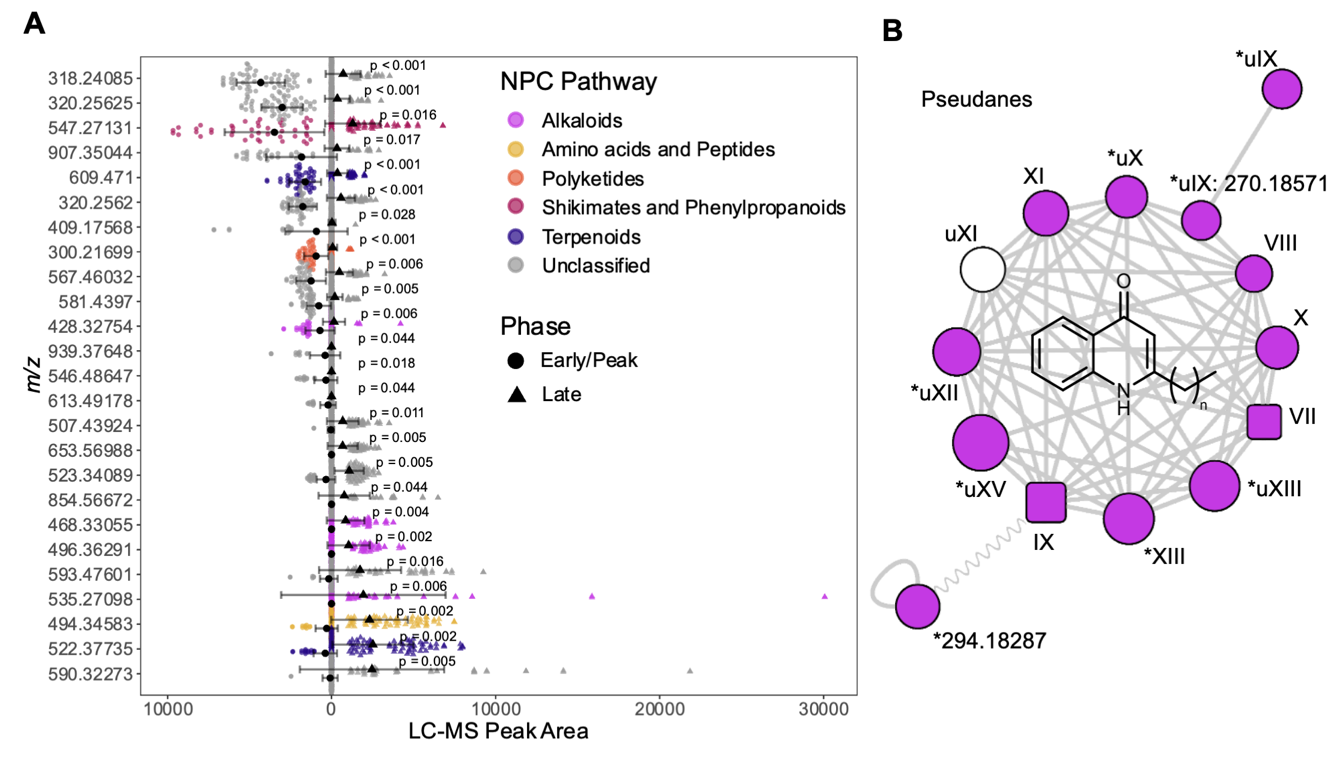** | |

**Supplementary Figure 6.** Metabolomic analysis **A)** Venn diagram of the total metabolic features shared between the three phases. **B)** Predicted amino acids and peptides (>300 m/z) features per day. Color denotes succession phase. Solid black line represents the mean and vertical colored line standard deviation (*N* = 9). **C)** GNPS generated a molecular network of the pseudanes family cluster annotated by SIRIUS detected at day 29 in a single replicate (peak phase). Squares represent GNPS spectral library hits, nodes with asterisks represent new derivatives detected in this dataset. Sinusoidal edges represent adduct connections between nodes. Nomenclature indicates alkyl chain length, monounsaturation (e.g. uIX) or metabolites not yet named.

##

**A**
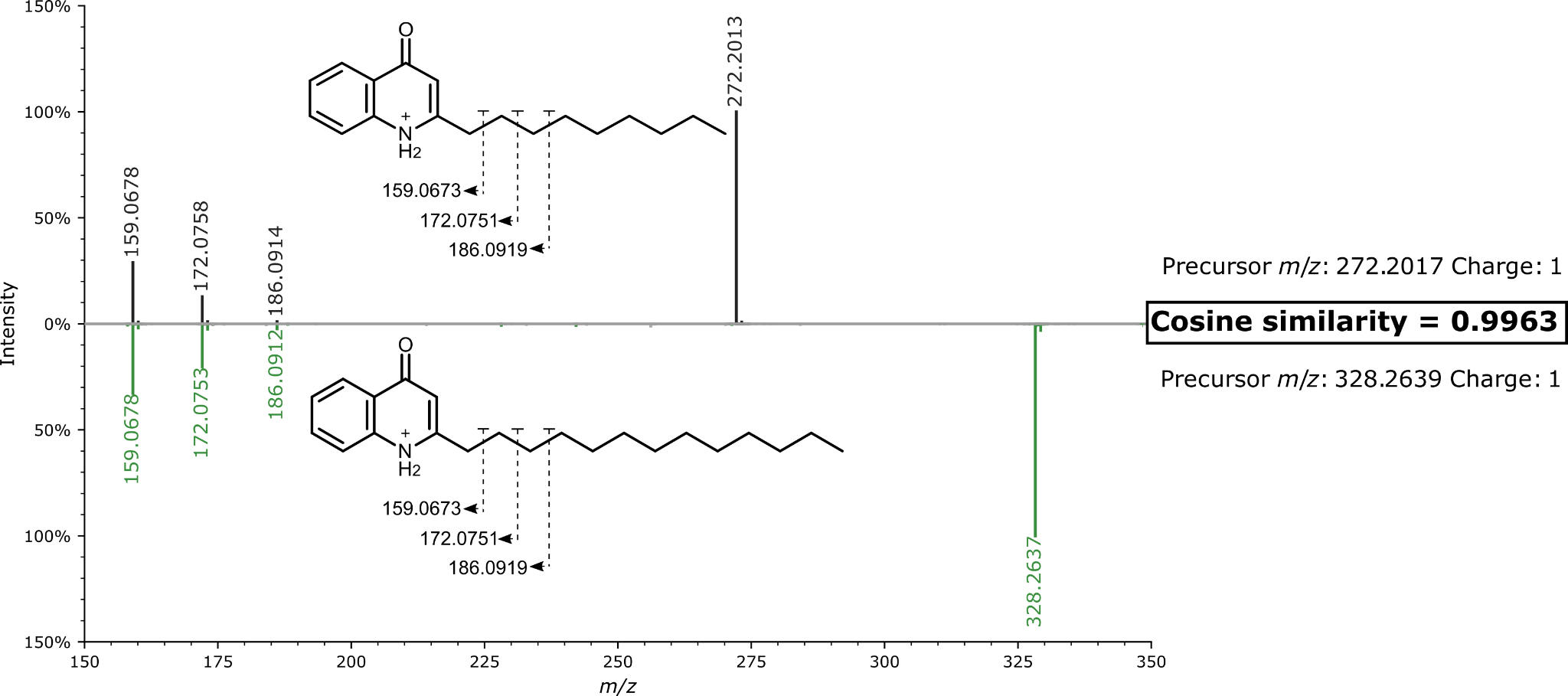
**B**
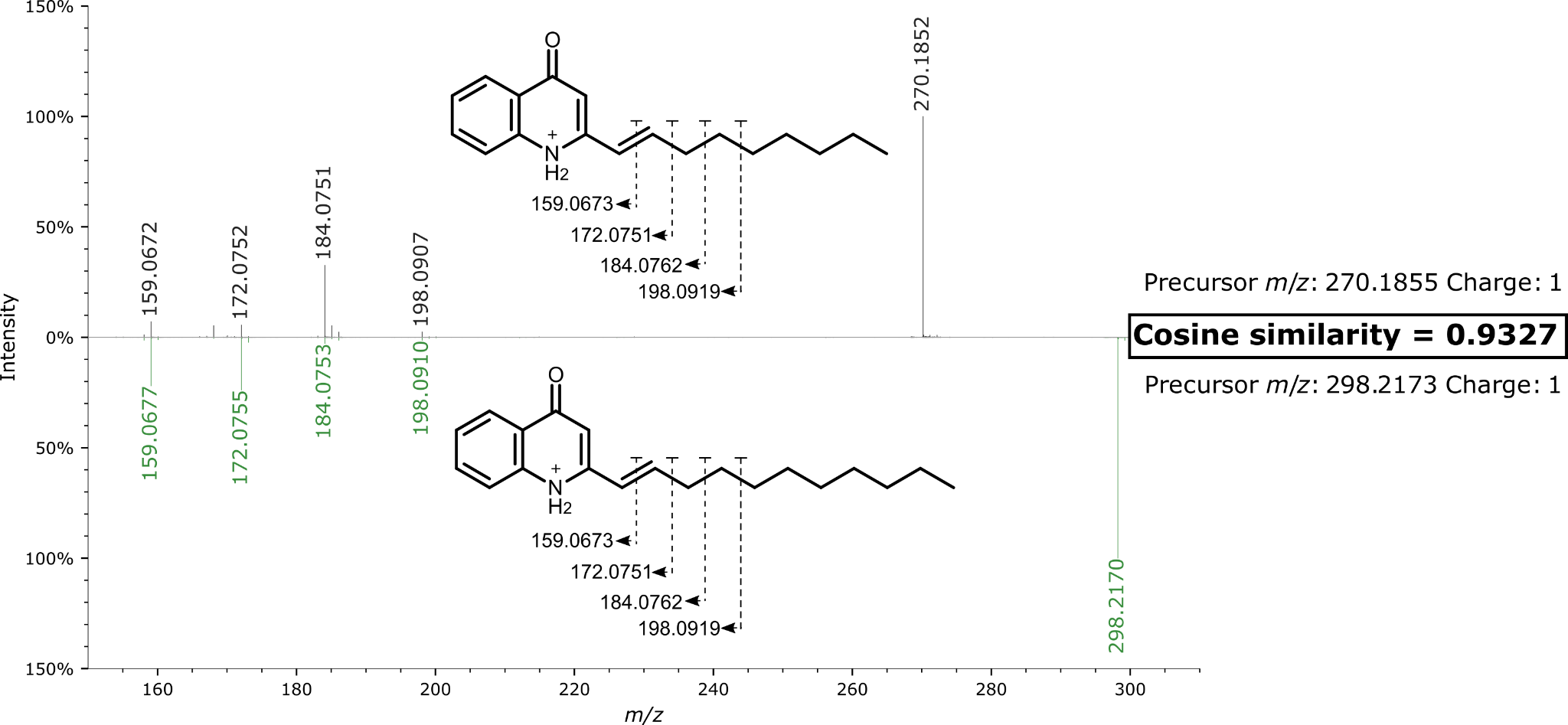


## **Supplementary Figure 7.** Fragmentation analysis for determination of new pseudane derivatives. All additional derivatives described in this study were analyzed in a similar manner to these two examples. **A)** Mirror plot (generated using Metabolomics USI [[3]](https://paperpile.com/c/WZ4GiO/PvQT)) of pseudane IX (*m/z* 272.2017) and pseudane XIII (*m/z* 328.2639). **B)** Mirror plot of pseudane uIX (*m/z* 270.1855) and pseudane uXI (*m/z* 298.2173). Green annotations indicate mirror matches corresponding to the top spectra. Fragment masses annotated to the structures are theoretical fragment masses generated in ChemDraw Professional 18.1

## **References**

1. [Riisgaard K, Swalethorp R, Kjellerup S, Juul-Pedersen T, Nielsen TG. Trophic role and top-down control of a subarctic protozooplankton community. Mar Ecol Prog Ser. 2014;500: 67–82. doi:](http://paperpile.com/b/WZ4GiO/zTPPO)[10.3354/meps10706](http://dx.doi.org/10.3354/meps10706)

2. [Bech PK, Lysdal KL, Gram L, Bentzon-Tilia M, Strube ML. Marine sediments hold an untapped potential for novel taxonomic and bioactive bacterial diversity. mSystems. 2020;5:e00782-20. doi:](http://paperpile.com/b/WZ4GiO/zER5f)[10.1128/mSystems.00782-20](http://dx.doi.org/10.1128/mSystems.00782-20)

3. [Bittremieux W, Chen C, Dorrestein PC, Schymanski EL, Schulze T, Neumann S, et al. Universal MS/MS visualization and retrieval with the metabolomics spectrum resolver web service. doi:](http://paperpile.com/b/WZ4GiO/PvQT)[10.1101/2020.05.09.086066](http://dx.doi.org/10.1101/2020.05.09.086066)
